# TRIM59 suppresses the brain ischaemia/reperfusion injury and pyroptosis of microglial through mediating the ubiquitination of NLRP3

**DOI:** 10.1038/s41598-024-52914-7

**Published:** 2024-01-30

**Authors:** Liangtian Zhang, Gang Li, Ying Li

**Affiliations:** 1 Department of Emergency Medicine, Chun’an First People’s Hospital, Hangzhou City, Zhejiang Province China; 2 Emergency and Critical Care Center, Department of Emergency Medicine, Zhejiang Provincial People’s Hospital (Affiliated People’s Hospital, Hangzhou Medical College), Hangzhou, Zhejiang China; 3 Department of Special Inspection, Hangzhou TCM Hospital, Affiliated to Zhejiang Chinese Medical University, No. 453, Tiyuchang Road, Hangzhou City, Zhejiang Province China

**Keywords:** Cell biology, Neuroscience, Biomarkers, Pathogenesis

## Abstract

Cerebral ischaemia/reperfusion (I/R) injury induces irreversible brain injury and causes functional impairment. Ubiquitination plays a crucial role in protein degradation, but its role in cerebral I/R injury remains unclear. Differentially expressed genes in stroke were identified by analysing the microarray dataset GSE119121. Cerebral I/R was simulated in vitro by treating human microglial HMC3 cells with oxygen–glucose deprivation/reperfusion (OGD/R). Cell viability was tested by Cell Counting Kit 8 (CCK-8) assays, and pyroptosis was examined by flow cytometry. Lactate dehydrogenase (LDH) and inflammatory cytokine secretion were measured by LDH cytotoxicity assays and enzyme-linked immunosorbent assay (ELISA), respectively. The cerebral I/R animal model was established by middle cerebral artery occlusion (MCAO) surgery in rats. Bioinformatic analysis indicated that tripartite motif-containing protein 59 (TRIM59) is downregulated in stroke, which was verified in cerebral I/R models. The upregulation of TRIM59 promoted viability and inhibited pyroptosis in OGD/R-treated microglia and alleviated cerebral I/R injury in vivo. TRIM59 attenuated NOD-like receptor family pyrin domain containing 3 (NLRP3) protein expression through ubiquitination, thus degrading NLRP3 and alleviating OGD/R-induced injury. TRIM59 relieves cerebral I/R injury in vivo and in vivo. Mechanistically, TRIM59 directly interacts with NLRP3 and inhibits NLRP3 through ubiquitination. Targeting the TRIM59/NLRP3 signalling axis may be an effective therapeutic strategy for cerebral I/R.

## Introduction

Stroke has been listed in the top 5 causes of death worldwide and is caused by a shortage of blood supply in the brain due to various causes. Stroke deprives neurons of glucose, oxygen and energy, leading to the death of neurons. In addition, the restoration of blood perfusion and reoxygenation can aggravate the injury to ischaemic cells, which is called cerebral ischaemia/reperfusion (I/R) injury^1,2^.

The pathological process of cerebral I/R injury involves excitatory amino acid toxicity, calcium overload, free radical damage, cytotoxic effects of nitric oxide and immune inflammation^3–6^. Inflammation is also related to the damage caused by cerebral I/R^7,8^. Microglia are considered to be the macrophages of the central nervous system and are the major resident immune cells involved in the inflammatory response^9,10^. Overactivated microglia lead to inflammation and aggravate tissue injury and neuronal death by inducing the excessive production of inflammatory cytokines^11^. Therefore, suppressing of the overactivation of microglia may be helpful to protect central nervous system.

Recently, microglial pyroptosis is a proinflammatory form of programmed cell death and has been reported to regulate stroke-induced inflammation^12^. Pyroptosis depends on the activation of inflammasomes. The oligomerization of NOD-like receptor family pyrin domain containing 3 (NLRP3) with the apoptosis-associated speck-like protein containing the CARD (ASC) and pro-Caspase-1 activates the NLRP3 inflammasome, leading to Caspase-1 cleavage^13,14^. Therefore, suppressing microglial pyroptosis might be a novel strategy for cerebral I/R.

Excessive activation of the NLRP3 inflammatory complex is significantly associated with a variety of inflammatory diseases, such as type 2 diabetes mellitus^15^, atherosclerosis^16^, Alzheimer's disease^17^ and cancer^18^. Recent studies have confirmed that activation of the NLRP3 inflammatory complex promotes the pathological development of cerebral I/R injury^19–21^. Additionally, the NLRP3 inflammasome can be activated by posttranslational modifications (PTMs), such as ubiquitination, sumoylation, alkylation, phosphorylation, and S-nitrosylation^22–24^. Ubiquitination has been proven to play an important regulatory role in NLRP3-mediated inflammation^25,26^. Ubiquitin is a protein composed of 76 amino acids that is typically bound to the lysine residues of substrate proteins through isopeptide bonds^27^. However, the role and mechanism of ubiquitination in pyroptosis and the progression of I/R injury remain unclear.

TRIM proteins participate in multiple cellular processes including cell proliferation, transcriptional regulation, immunity and cancer progression^28,29^. A series of studies indicated that many TRIM proteins are linked to pyroptosis^30,31^. TRIM59 is a member of the TRIM protein superfamily, and has a TRIM or RBCC motif consisting of a RING-finger domain (R), a B-box domain (B), and a coiled-coil domain (CC). Increasing evidence showed that TRIM59 has been identified as contributing to tumor progression^32^. In the current study, we aimed to analyse the role of TRIM59 in cerebral I/R injury. Whether the ubiquitination of NLRP3 mediated by TRIM59 is related to pyroptosis in cerebral I/R was also investigated. Our findings will provide new strategies for the treatment of stroke.

## Materials and methods

### Bioinformatic analysis

GSE119121 is an expression profile of the blood of rats with stroke. As described in Dagonnier M et al., 0-h and 2-h data were selected for analysis to obtain genes that were abnormally expressed in rats with stroke^33^. The criteria for gene screening were as follows: *p* < 0.05 and absolute value of log2 (fold change) > 1.

### Cell culture and oxygen–glucose deprivation/reperfusion (OGD/R) model establishment

Human microglia HMC3 were obtained from ATCC (Manassas, VA, USA) and cultured in DMEM (Gibco, Grand Island, NY,USA) with 10% FBS (Gibco), 100 U/mL penicillin (Gibco), and 100 μg/mL streptomycin (Gibco). HMC3 cells were tested for mycoplasma and authenticated by Procell Life Science & Technology Co., Ltd. (Wuhan, China) to exclude contamination with other cell lines or incorrect identification. Oxygen–glucose deprivation/reperfusion (OGD/R) was used to establish a cell model of cerebral I/R injury in vitro as previously described^34^. Briefly, HMC3 cells were cultured in Earle’s balanced salt solution (Gibco) at 37 °C with 95% N_2_ and 5% CO_2_. Two hours later, the medium was replaced with normal medium, and the cells were cultured normally.

### Cell transfection

pcDNA3.1-TRIM59 (TRIM59), pcDNA3.1-NLRP3 (NLRP3), and corresponding controls (Vector) were purchased from GenePharma (Shanghai, China). HMC3 cells were seeded into six-well plates (3 × 10^5^/mL) and transfected using Lipofectamine^®^ 2000 reagent (Invitrogen, CA, USA). After 48 h, the cells were collected for OGD/R inducation and subsequent experiments.

### Production of lentiviral vectors

Lentiviral particles overexpressing TRIM59 (LV-TRIM59) and the negative controls (LV-NC) were obtained from GenePharma and transfected into HEK293T cells. HEK293T culture medium was collected at 48 h after transfection.

### Animals and induction of MCAO

The rat experiments were carried out in compliance with the revised Animals (Scientific Procedures) Act 1986 and the ARRIVE guidelines (http://www.nc3rs.org.uk/page.asp?id=1357). In addition, the rat experiments were approved by the Animal Ethical and Welfare Committee (approval no. MDKN-2021–058). Twenty-four SPF healthy SD rats weighing 260 ± 10 g were purchased from Shanghai Slack Laboratory Animal Co., Ltd. (Shanghai, China). The production licence number was SCXK (Shanghai) 2014–0002, and the qualification licence number was 2015000518015. All animal experiments complied with ethical requirements. The rats were randomly divided into four groups, with 6 rats in each group: sham operation group (Sham), model group (MCAO), TRIM59 overexpression model group (MCAO + LV-TRIM59) and TRIM59 overexpression negative control model group (MCAO + LV-NC).

To establish MCAO in rats, the rats were anaesthetized with 2% sodium pentobarbital (2 mL/kg) after 12 h of fasting. The common carotid artery, external carotid artery and internal carotid artery were separated, a small incision was made in the common carotid artery, a thread plug was inserted, and the internal carotid artery was ligated. After 2 h of obstruction, the 4–0 (0.26 mm diameter) wire plug was removed to achieve reperfusion. The sham group underwent the same operations as the MCAO group except that the wire plug was not inserted. After the rats were awake, neurological impairment was scored according to a previous report^35^.

### Stereotaxic injection of the lentivirus

LV-TRIM59 and LV-NC were injected into the left lateral ventricle one month before MCAO surgery to overexpress TRIM59 in rats. After anaesthesia by intraperitoneal injection of 1% pentobarbital sodium (40 mg/kg), the rats were fixed with a brain stereoscopic locator, and the left cerebral cortex was positioned using the following coordinates relative to the bregma: 1.0 mm posterior, 2.0 mm lateral, 3.0 mm below the horizontal plane of the bregma. The hole was drilled, and 2 μL lentivirus was injected at a depth of 1.2 mm with a needle. The needle was kept for 10 min after slow injection.

### Measurement of brain infarct volume

After the evaluation of neurological impairment, the rats were euthanized with 400 mg/kg pentobarbital sodium. The euthanasia and anaesthesia methods were carried out in accordance with the guidelines of the American Veterinary Medical Association. Then, the brains were immediately collected and cut into six coronal slices with a thickness of 2 mm. Then, the slices were dyed using 2% 2,3,5-triphenyltetrazolium chloride (TTC; BIOPIKE, Guangzhou, China) at 37 °C for 10 min. Next, brain infarct size was calculated by the following formula: (volume of total brain tissue-volume of total ipsilateral hemispheric damage)/volume of total brain tissue × 100.

### Nissl staining

The paraffin sections were dewaxed and stained with 10% methylene blue solution for 20 min. After being washed, the sections were treated with 95% alcohol, dehydrated, made transparent, sealed, and placed under an ordinary light microscope (Leica Microsystems, Wetzlar, Germany) for observation and photometry, and the number of intact neurons was counted. The total neuronal cells were divided into 10 areas and calculated, and then the degree of ischaemia-damaged neurons was calculated according to the percentage of damaged neurons in the total neurons^35^.

### Immunofluorescence analysis

Brain sections were fixed with 4% paraformaldehyde and then incubated in PBST containing 5% BSA (Santa Cruz, CA, USA) for 0.5 h. Next, the sections were treated with primary antibodies, including anti-IBA-1 (highly expressed in microglia) and anti-NLRP3, at 4 °C overnight, followed by incubation with the corresponding secondary antibody at 37 °C for 1 h. The nuclei were stained with DAPI (Beyotime, Shanghai, China). Finally, the sections were observed with a scanning fluorescence microscope (Leica Microsystems, Wetzlar, Germany) and analysed using ImageJ software (ver. 1.45r, National Institutes of Health, Bethesda, MD, USA).

### Real-time quantitative polymerase chain reaction (RT–qPCR)

The mRNA expression of TRIM59 and NLRP3 in brain tissues and HMC3 cells was assessed by RT-qPCR. We extracted total RNA using TRIzol^®^ reagent (Thermo Fisher, Waltham, MA, USA) and then reverse transcribed the RNA into cDNA (Hi-Fi cDNA Synthesis Kit, Abcam, Cambridge, MA, USA). RT-qPCR was performed using the SYBR Green Mix kit (Beyotime) in an Eppendorf Thermol Cycler system (Eppendorf AG, Shanghai, China). The target amplification process was performed as follows: predenaturation at 95 °C for 5 min, denaturation at 95 °C for 10 s, annealing at 60 °C for 20 s, and then extension at 72 °C for 30 s, all for 40 cycles. Quantitative analysis was then performed using the 2^-ΔΔCt^ method. The primer sequences are listed in Table 1.

**Table 1:** Primer sequences.

Primer	Forward (5′ → 3′)	Reverse (5′ → 3′)
TRIM59	ATGCACAATTTTGAGGAGGAG	ATGCACAATTTTGAGGAGGAG
NLRP3	CGCGTAGCACCATCTGAAAT	AGTGCAGGGTCCGAGGTATT
GAPDH	CAAGATCATCAGCAATGCC	CTGTGGTCATGAGTCCTTCC

### Cell viability assay

Cell counting kit 8 (CCK-8, Beyotime) was used to evaluate the viability of HMC3 cells. In brief, the cells were seeded into 96-well plates, and 10 μL/well CCK-8 reagent was added. After 4 h of culture, cell viability was detected at an absorbance of 450 nm with a microplate reader (Bio-Rad).

### Lactate dehydrogenase (LDH) cytotoxicity evaluation

To evaluate cell damage and measure cell death, LDH release by HMC3 cells was determined by an LDH Cytotoxicity Assay Kit (Beyotime). In brief, HMC3 cells were seeded into a 96-well plate and incubated with the LDH reagent for 0.5 h to measure LDH release. The absorbance values were obtained at 490 nm with a microplate reader (Bio-Rad, Hercules, CA, USA).

### Enzyme-linked immunosorbent assay (ELISA)

The levels of interleukin-1β (IL-1β) and IL-18 in HMC3 cell lysates were measured by ELISA using a human IL-1β ELISA kit (ab214025, Abcam) and a human IL-18 ELISA kit (ab215539, Abcam) according to the manufacturer's instructions. IL-1β and IL-18 levels in rat serum were detected by a rat IL-1β ELISA kit (ab100768, Abcam) and a rat IL-18 ELISA kit (ab213909, Abcam), respectively.

### Measurement of pyroptosis

Flow cytometry was performed to examine pyroptosis. HMC3 cells were digested with trypsin and washed with PBS. Pyroptosis was assessed with FLICA 660-YVADFMK in accordance with the manufacturer's protocol. The cells were stained with PI to identify cells with membrane pores and analysed by a BD-Verse flow cytometer (BD, NJ, USA).

### Western blot analysis

HMC3 cells were collected to extract proteins (RIPA, Beyotime), and the protein concentration was determined using the BCA method. Then, the proteins were separated by 10% SDS‒PAGE and transferred to PVDF membranes. After the membrane was transferred, the proteins were blocked in 5% skim milk at room temperature for 2 h. Diluted primary antibodies (Table 2) were added and incubated overnight at 4 °C. HRP-labelled secondary antibodies were then added and incubated. Finally, the protein bands were visualized using an ECL system (Thermo Fisher).

**Table 2:** Antibodies.

Target	Dilution	Source	Species specificity	Catalogue number	Manufacturer
TRIM59	1/600	Rabbit	Mouse, rat, human	ab69639	Abcam
NLRP3	1/500	Rabbit	Human	ab263899	Abcam
NLRP3	1/100	Rabbit	Mouse, rat, human	AF2155	Beyotime
Cleaved-caspase-1	1/500	Rabbit	Human	ab207802	Abcam
GSDMD-N	0.2 µg/mL	Rabbit	Human	ab9722	Abcam
Flag-tag	1.5 mg/mL	Rabbit	All	F7425	Sigma
HA-tag	0.8 μg/mL	Rabbit	All	H6908	Sigma
GAPDH	1/2500	Rabbit	Mouse, human	ab9485	Abcam
HRP anti-rabbit IgG antibody	1/2000	Goat	Rabbit	ab288151	Abcam
IBA-1	1/2000	Rabbit	Mouse, rat, human	ab178846	Abcam

### Protein stability assessment

A cycloheximide (CHX) blocking assay was performed to determine the half-life of NLRP3 as previously reported^36^. HMC3 cells were incubated with CHX (100 μg/mL, Sigma-Aldrich, St. Louis, MO,USA), and the protein level of NLRP3 was detected at different time points (0, 2, 4, 8 h) after CHX treatment.

### Coimmunoprecipitation (Co-IP)

HMC3 cells were lysed in RIPA lysis buffer (Beyotime). A 1/10 volume of supernatant was collected as the input, and half of the remaining supernatant was incubated with 20 µL/mL protein A/G Sepharose beads (Beyotime) at 4 °C for 1 h to remove nonspecific hybrid proteins. Afterwards, the lysates were incubated with 2 µg anti-NLRP3 antibodies (ab263899, Abcam)/anti-TRIM59 antibodies (ab69639, Abcam) or negative control IgG (Beyotime) at 4 °C overnight and then rotated at 4 °C with a mixture of protein A/G Sepharose beads (20 µL/mL) for 4 h. The bound proteins were analysed by western blotting.

### Ubiquitination assay

The cells were transfected with vectors expressing Flag-NLRP3, HA-UB, TRIM59 and the control. Whole-cell extracts were immunoprecipitated with anti-HA and analysed by immunoblotting with anti-Flag antibodies.

### Statistical analysis

Each experiment was repeated three times, and all data are expressed as the mean ± SD and were calculated by GraphPad Prism 8.3. Student’s t test was used to analyse the differences between two groups, and one-way analysis of variance (ANOVA) was performed for comparisons between multiple groups. All results were considered significantly different when *p* < 0.05.

### Ethics approval

The rat experiments were approved by the Animal Ethical and Welfare Committee (approval no. MDKN-2021-058). All animal experiments complied with ethical requirements.

## Results

### TRIM59 is expressed at low levels in cerebral I/R and OGD/R models

**Figure 1:**
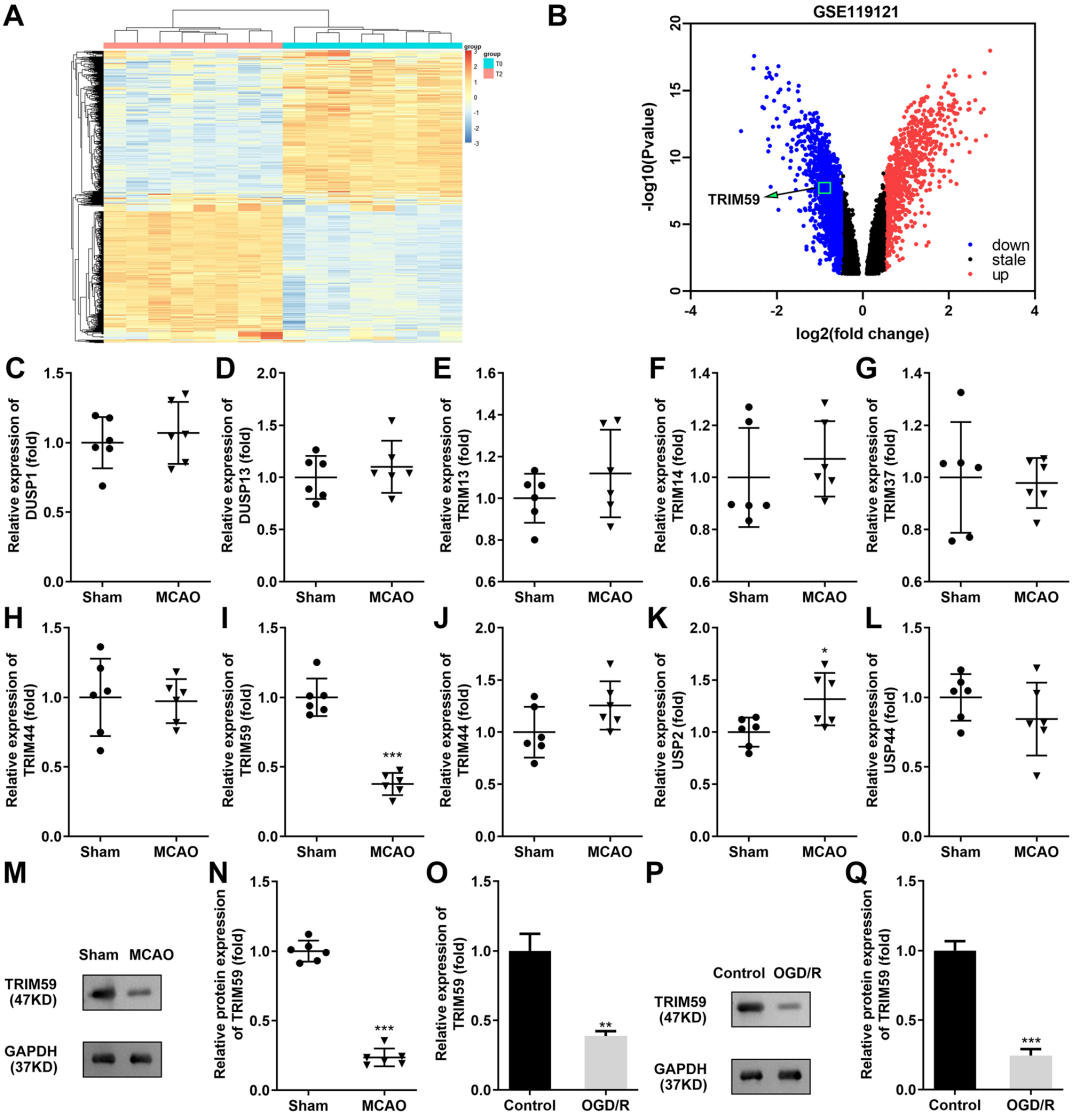
TRIM59 is expressed at low levels in a cerebral I/R animal model and cell model. (**A**) Heatmap analysis of differentially expressed genes in stroke from the microarray expression profiling dataset GSE119121. (**B**) A total of 344 aberrantly upregulated genes and 267 downregulated genes were identified. (**C**–**L**) The mRNA levels of ten ubiquitination-related genes in MCAO rats were detected by RT-qPCR, n = 6. (**M**, **N**) The protein expression of TRIM59 in the brain tissues of rats was detected by western blotting, n = 6. The mRNA (**O**) and protein expression (**P**, **Q**) of TRIM59 in HMC3 cells were evaluated by RT-qPCR and western blot analysis, respectively. n = 3. **p < 0.01, ***p < 0.001.

Differentially expressed genes in the blood samples of 8 spontaneously hypertensive rats at 0 and 2 h post stroke induction by MCAO were identified (Fig. 1A). As indicated in Fig. 1B, 344 aberrantly elevated genes and 267 downregulated genes were identified. Ten ubiquitination-related genes that were significantly downregulated in MCAO rats were screened (Fig. 1C–L), among which TRIM59 was selected as the most downregulated gene (Fig. 1I). Then, the low protein expression of TRIM59 was verified in the brain tissues of MCAO rats, which were established to mimic cerebral I/R in vivo (Fig. 1M, N). The OGD/R model in HMC3 cells was established, and TRIM59 mRNA and protein expression was shown to be dramatically downregulated (Fig. 1O, Q).

### TRIM59 protects microglia from OGD/R injury

**Figure 2:**
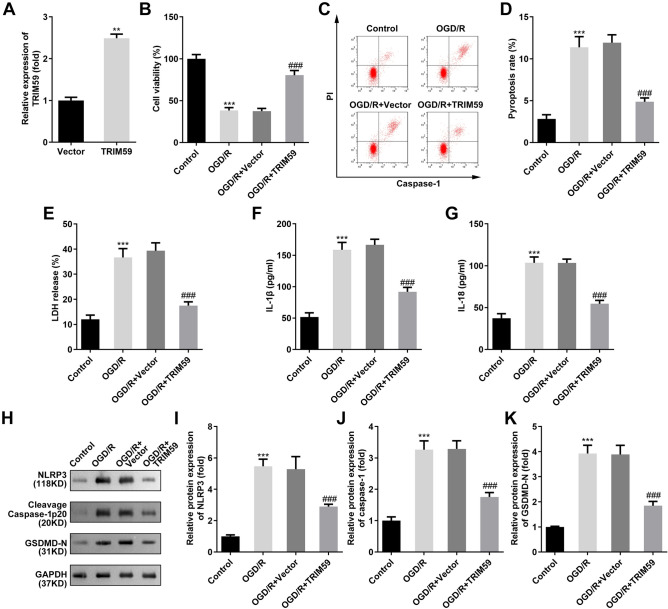
(**A**) TRIM59 protects microglia from OGD/R injury. TRIM59 expression levels were detected by RT-qPCR after transfection with TRIM59 overexpression vectors, n = 3. (**B**) Cell viability was detected by CCK-8 assays two houres after OGD/R performace. (**C**, **D**) Pyroptosis in HMC3 cells was measured by flow cytometry. (**E**) The release of LDH was examined 48 h after transfection. (**F**, **G**) ELISA was used to evaluate the levels of IL-1β and IL-18. (**H**–**K**) Western blotting was used to detect the protein levels of NLRP3, cleaved Caspase-1 p20 and GSDMD-N in HMC3 cells. n = 3, **p < 0.01, ***p < 0.001, versus Vector or control group. ###*p* < 0.001, versus OGD/R + Vector group.

Then, the role of TRIM59 was studied using the cerebral OGD/R model. TRIM59 mRNA expression was significantly upregulated in HMC3 cells after transfection (Fig. 2A). A two-hour OGD/R treatment significantly reduced the viability of HMC3 cells (Fig. 2B). The pyroptosis rate of HMC3 cells was detected by flow cytometry (Fig. 2C, D). OGD/R treatment accelerated pyroptosis in HMC3 cells, which was further confirmed by the increases in LDH, IL-1β and IL-18 (Fig. 2E–G). Moreover, NLRP3, cleaved Caspase-1 p20, and GSDMD-N levels were upregulated in OGD/R-treated HMC3 cells (Fig. 2H–K). The upregulation of TRIM59 significantly alleviated OGD/R-induced injury by promoting cell growth and inhibiting pyroptosis (Fig. 2B–K).

### TRIM59 attenuates NLRP3 protein expression through ubiquitination

**Figure 3:**
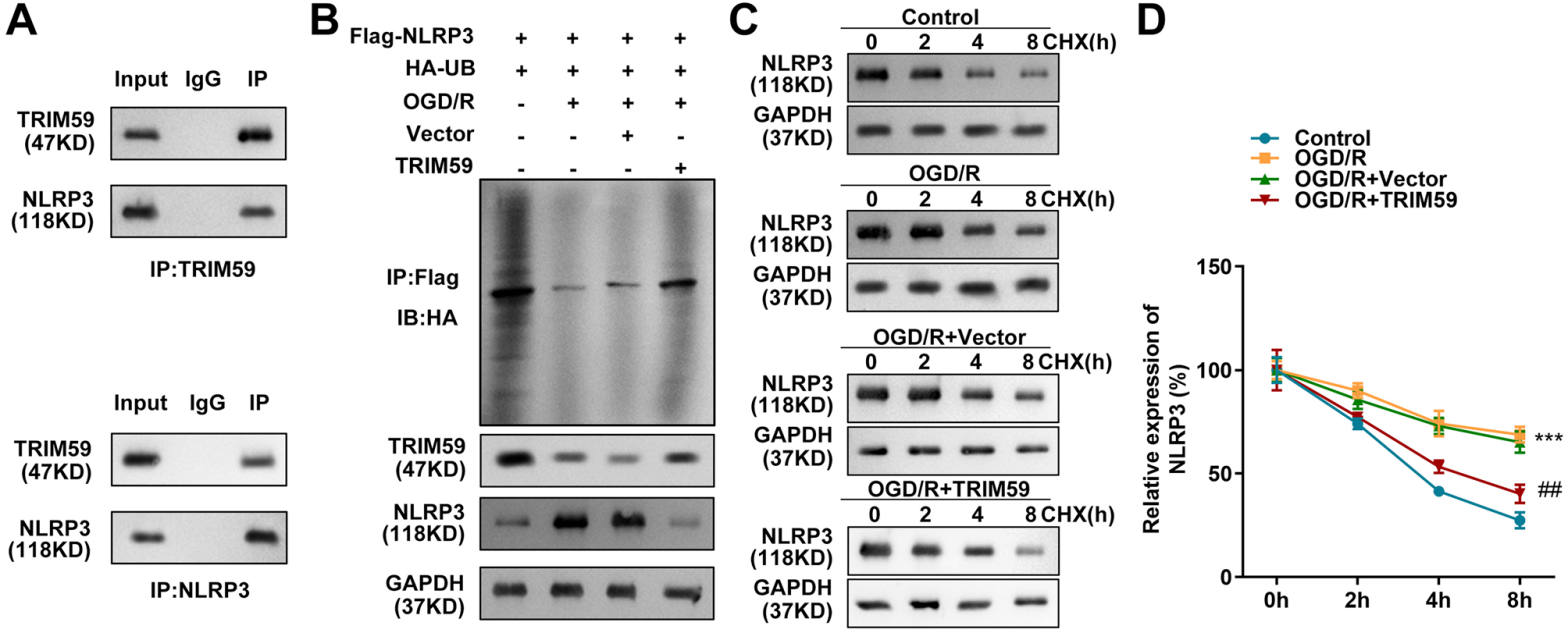
(**A**) TRIM59 attenuates NLRP3 protein expression through ubiquitination. The interaction between TRIM59 and NLRP3 was detected by Co-IP. (**B**) After transfection, IP using anti-Flag was performed. The ubiquitination and expression of HA in the pull-down product was detected by western blotting. (**C**, **D**) Western blot analysis of the half-life of NLRP3 protein in HMC3 cells under OGD/R conditions and with TRIM59 overexpression. n = 3, ***p < 0.001, versus control group. ##*p* < 0.01, versus OGD/R + Vector group.

As an E3 ubiquitin ligase, TRIM59 has ubiquitin protein transferase activity. Therefore, TRIM59 was thought to modulate NLRP3 stability through the ubiquitin‒proteasome pathway. The co-IP results revealed the interaction between TRIM59 and NLRP3 (Fig. 3A). Then, a ubiquitination assay was performed on HMC3 cells after transfection. As shown in Fig. 3B, OGD/R knocked down the protein expression of TRIM59 and decreased the ubiquitination of NLRP3. However, the upregulation of TRIM59 enhanced the ubiquitination of NLRP3 and inhibited NLRP3 protein levels (Fig. 3B), suggesting that TRIM59 might affect the stability of NLRP3. To substantiate this conclusion, we treated HMC3 cells with OGD/R and the protein synthesis inhibitor CHX. The results suggested that OGD/R extended the half-life of the NLRP3 protein, while overexpression of TRIM59 shortened the half-life of the NLRP3 protein in HMC3 cells after CHX treatment (Fig. 3C, D).

### NLRP3 reverses the TRIM59-mediated protection against OGD/R injury

**Figure 4:**
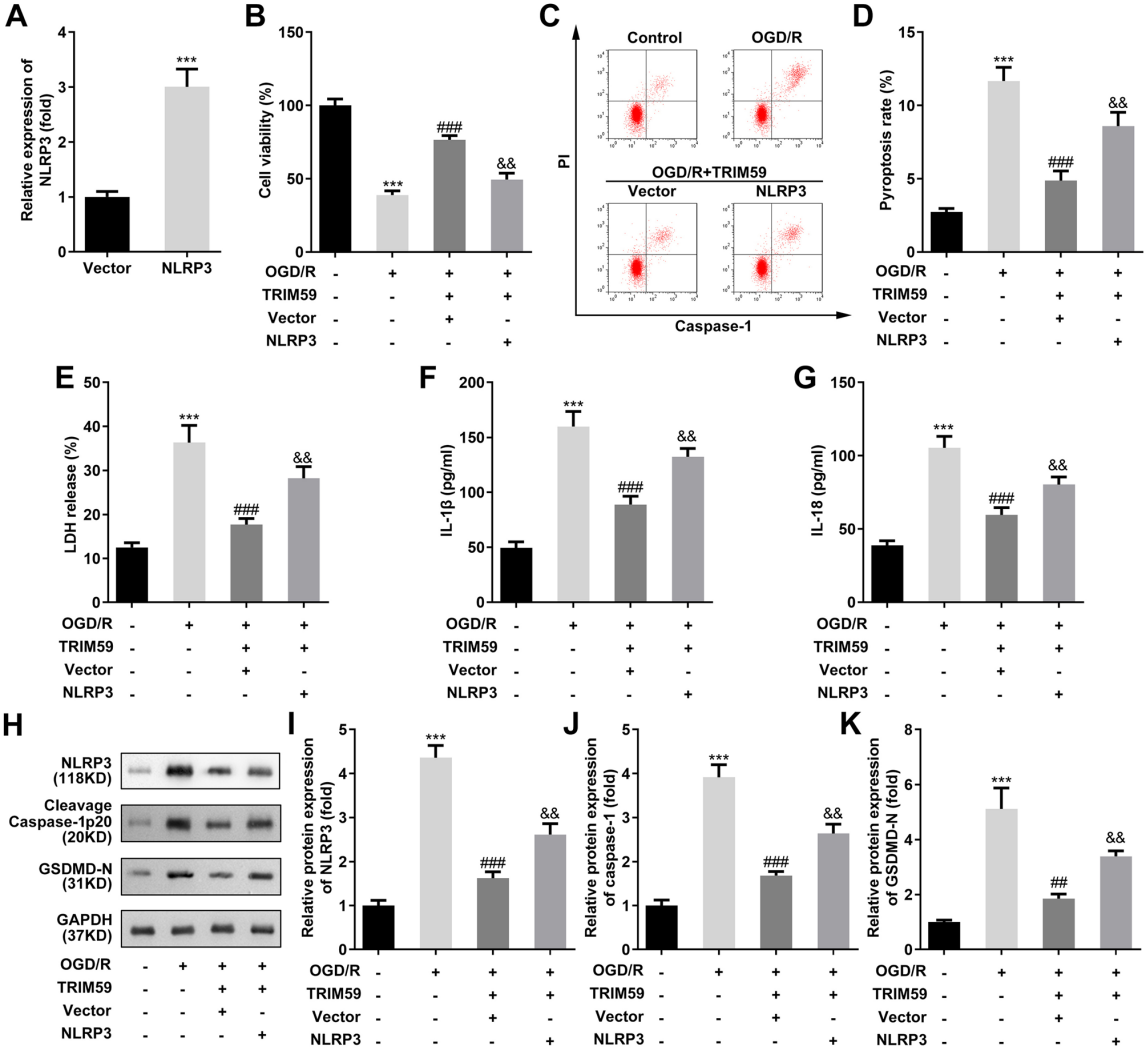
NLRP3 is required for TRIM59-mediated suppression of OGD/R injury. (**A**) NLRP3 expression levels were detected by RT-qPCR after transfection with NLRP3 vectors. (**B**) Cell viability was detected by CCK-8 assays two houres after OGD/R performace. (**C**, **D**) Pyroptosis in HMC3 cells was measured by flow cytometry. (**E**) The release of LDH was examined 48 h after transfection. (**F**, **G**) ELISA was used to evaluate the levels of IL-1β and IL-18. (**H**–**K**) Western blotting was used to detect the protein levels of NLRP3, cleaved Caspase-1 p20 and GSDMD-N in HMC3 cells. ***p < 0.001, versus Vector or control group. n = 3, ###*p* < 0.001, versus OGD/R group. &&*p* < 0.01, versus OGD/R + TRIM59 + Vector group.

To further assess whether TRIM59 protects HMC3 cells from OGD/R injury via NLRP3, NLRP3 was elevated in TRIM59-overexpressing cells (Fig. 4A). CCK-8 and caspase-1/PI apoptosis assays demonstrated that the upregulation of NLRP3 prominently neutralized the effects of TRIM59 overexpression on decreasing viability and increasing pyroptosis in HMC3 cells treated with OGD/R (Fig. 4B, D). Moreover, LDH, IL-1β and IL-18 concentrations that were decreased by TRIM59 overexpression were dramatically decreased by the upregulation of NLRP3 (Fig. 4E–G). The protein levels of NLRP3, cleaved Caspase-1 p20, and GSDMD-N were markedly enhanced by NLRP3 overexpression due to TRIM59 overexpression (Fig. 4H–K).

### TRIM59 alleviates cerebral I/R injury in vivo

**Figure 5:**
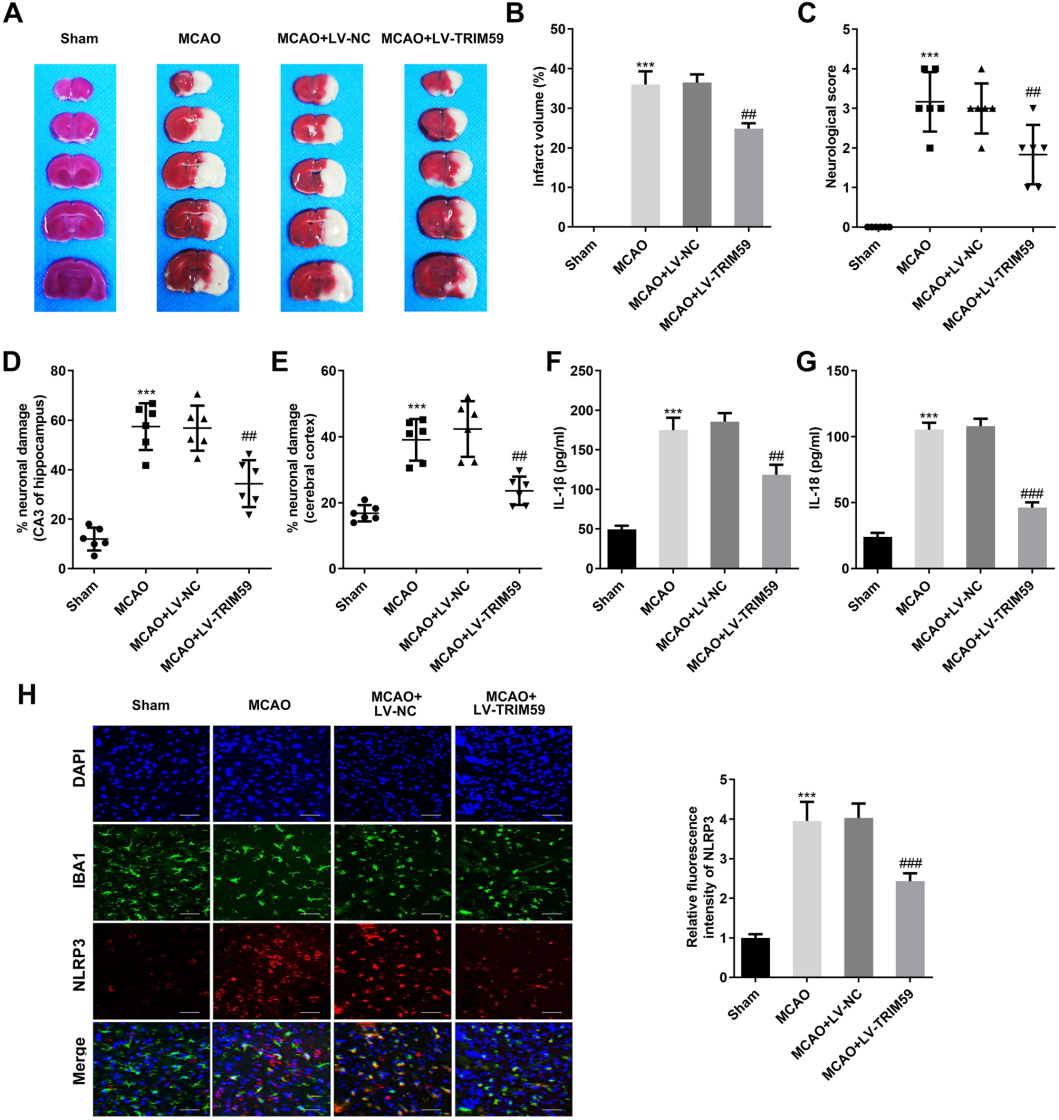
TRIM59 alleviates cerebral I/R injury in vivo. Images of TTC-stained complete coronal sections in the MCAO and sham groups. (**B**) The infarct volume was determined. (**C**) Neurologic scores in rats of the MCAO and Sham groups were evaluated. (**D**) Quantitative evaluation of the neuronal damage rate of the hippocampal CA3 region and (**E**) cerebral cortex was performed. (**F**, **G**) The expression of IL-18 and IL-1β in the serum of rats was detected by ELISA. (**H**) Immunofluorescence analysis was performed to evaluate the localization and expression of IBA1 and NLRP3. n = 6, ***p < 0.001, versus Sham group. ##*p* < 0.01, ###*p* < 0.001, versus MCAO + LV-NC group.

The neurological score and infarct volume were evaluated 7 days after MCAO. The infarct volume was prominently elevated in the MCAO group, but there was almost no infarct volume in the sham-operated group. The increase in TRIM59 notably decreased the infarct volume in MCAO rats (Fig. 5A, B). Moreover, the neurological score was prominently enhanced in MCAO-treated rats, while these effects were suppressed after TRIM59 upregulation (Fig. 5C). The Nissl staining results confirmed that MCAO dramatically aggravated neuronal injury in rats, and shrunken nuclei and disorderly arrays were observed in model rats (Fig. 5D, E). Additionally, MCAO prominently promoted the levels of IL-1β and IL-18 in rat serum, while TRIM59 dramatically inhibited this increase (Fig. 5F, G). Furthermore, the immunofluorescence results demonstrated that NLRP3 expression was elevated in the MCAO group, and TRIM59 overexpression significantly downregulated NLRP3 levels (Fig. 5H).

## Discussion

TRIM59 was downregulated in the brain tissues of MCAO rats and OGD/R-treated HMC3 cells. Our data suggested that the upregulation of TRIM59 promoted cell viability and inhibited pyroptosis in microglia after OGD/R treatment. Moreover, TRIM59 directly interacted with NLRP3 and inhibited NLRP3 through ubiquitination. These findings indicated that TRIM59 could alleviate cerebral I/R injury by inhibiting NLRP3 expression.

Cerebral I/R injury refers to injury caused by insufficient blood supply to the brain tissue and the restoration of blood perfusion. This process triggers an innate immune response and leads to an inflammatory cascade^19^. Inhibiting NLRP3 inflammasome activation has been used as one of the therapeutic methods for cerebral I/R^21,37^. For example, ibrutinib, a Bruton's tyrosine kinase inhibitor, can inhibit NLRP3 inflammasome, caspase-1 and IL-1β activation, thereby alleviating cerebral I/R injury^38^.

Ubiquitination can affect the biological functions of cells by regulating the stability and expression level of intracellular proteins, including cell proliferation, cell cycle, apoptosis, tumour development, metastasis, and invasion^39^. Changes in protein stability and abnormal expression are related to the development of diseases, and so the study of protein ubiquitination mechanisms can better explain the molecular mechanisms of diseases^40,41^. Ubiquitination was shown to modulate the stability of the NLRP3 inflammasome^42,43^. At present, only E3 ubiquitin ligases have been shown to negatively regulate the inflammasome by targeting NLRP3^44^. One of the main mechanisms involves controlling NLRP3 levels through the proteasome degradation pathway. Recently, TRIM59 has been identified as a novel molecular biomarker in various tumours^45^. TRIM59 promotes lung cancer progression by regulating autophagy or the NLRP3 inflammasome signalling pathway^28,46^. TRIM59 participates in tumour disease progression by regulating the ubiquitination of genes^47–49^. The presence of the unique N-terminal RING domain enables most TRIM family members to have extensive ubiquitination enzymatic activities and induce target proteins to enter the proteasome-dependent ubiquitination degradation process by binding them to ubiquitin. TRIM59 has the conserved RBCC domain of typical TRIM family proteins^28,46^. In addition to the three typical RBCC domains (RING finger domain, B-box domain and coiled-coil domain), the C segment of TRIM59 also has a TM domain that may be involved in the transmembrane localization of TRIM59, which is involved in regulating the intracellular localization of TRIM59. In the current work, bioinformatic analysis was carried out to screen genes in stroke related to ubiquitination, and TRIM59 was shown to be expressed at low levels in cerebral I/R animal and cell models. An increase in TRIM59 dramatically protected HMC3 cells from OGD/R injury. Afterwards, TRIM59 was found to regulate the ubiquitination of NLRP3.

Microglia, the resident immune cells tiling the whole CNS, exhibit an amazing capacity for regeneration^50,51^. Even when depleted in large quantities in the uninflamed central nervous system, microglia are able to quickly refill and return to normal density within a few days through the proliferation of residual cells. After neurodegeneration, they proliferate and accumulate in the diseased central nervous system^52^. Therefore, if the microglia are protected well, it may be possible to convert them into neurons to repair the damaged central nervous system where neurons are lost. In this study, TRIM59 promotes microglial survival and reduces infarction size and neuronal damage. Whether the protective mechanism benifits neurons should be investigated in the future. Meanwhile, which amino acid residue of NLRP3 is ubiquitination modified has not been elucidated.

Therefore, it is suggested that TRIM59 may act as a ubiquitin ligase to induce the ubiquitination-mediated degradation of NLRP3. Our data revealed that TRIM59 binds endogenously to NLRP3 and attenuates NLRP3 protein expression through ubiquitination. Furthermore, the half-life of the NLRP3 protein was decreased in TRIM59-overexpressing cells. These results suggest that the overexpression of TRIM59 contributes to NLRP3 protein degradation in microglia.

## Conclusion

Overall, TRIM59 relieves cerebral I/R injury in vivo and in vitro. Mechanistically, TRIM59 directly interacts with NLRP3 and inhibits NLRP3 through ubiquitination. The TRIM59/NLRP3 signalling axis may be a promising prognostic factor and a valuable therapeutic target for cerebral I/R (Supplementary Information).

### Supplementary Information

Supplementary Information 1.

Supplementary Information 2.

## Data Availability

The datasets used and analysed during the current study are available from the corresponding author on reasonable request.
