# Supplementary material for: TRIM59 suppresses the brain ischaemia/reperfusion injury and pyroptosis of microglial through mediating the ubiquitination of NLRP3
Source: Sci Rep. 2024 Jan 30;14:2511. doi: 10.1038/s41598-024-52914-7 (PMC10828378; doi:10.1038/s41598-024-52914-7)
Supplement: Supplementary file 1 — Supplementary Information 1. [file 41598_2024_52914_MOESM1_ESM.docx]

Fig.1M

|  | Sham | MCAO |
| --- | --- | --- |
| TRIM59 | 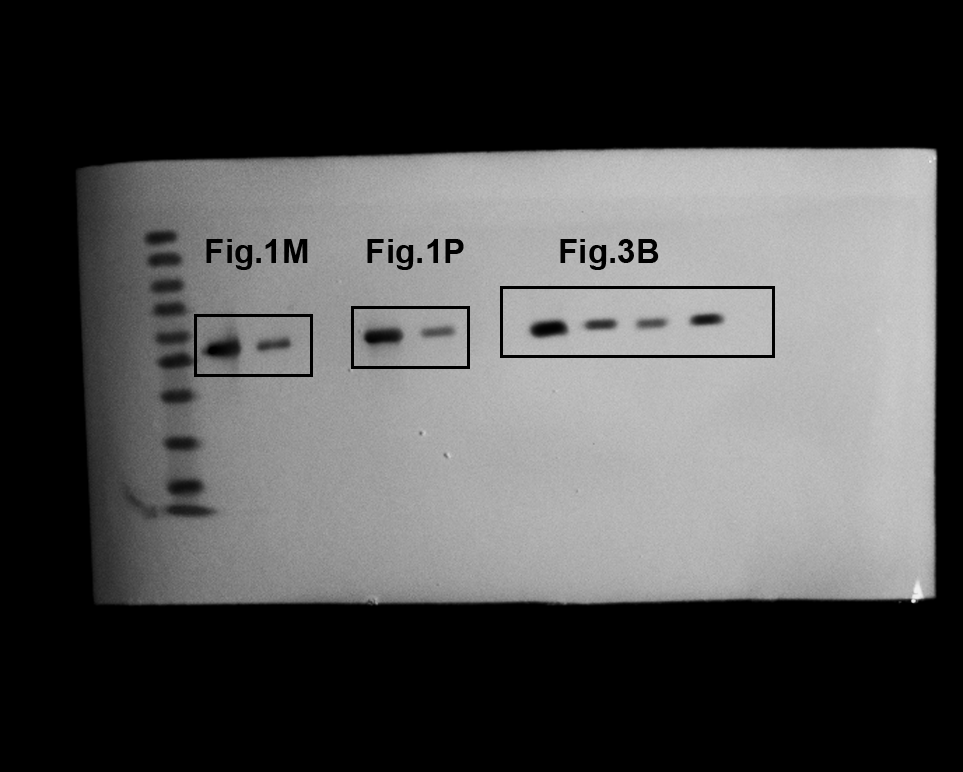 | |
| GAPDH | 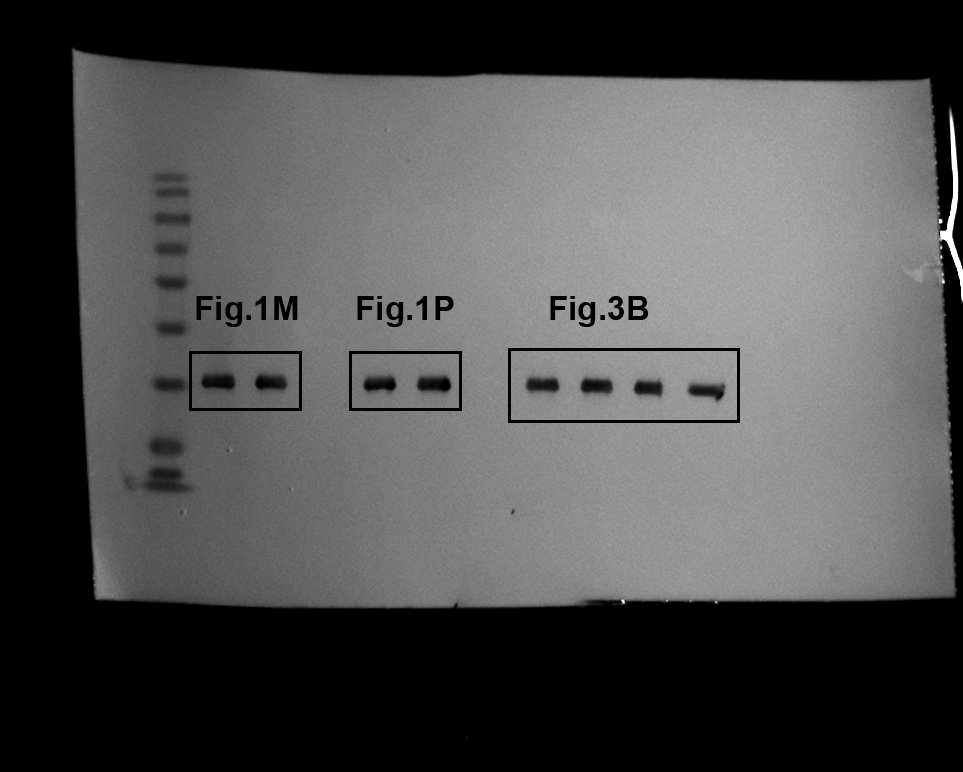 | |

Fig.1P

|  | Control | OGD/R |
| --- | --- | --- |
| TRIM59 | 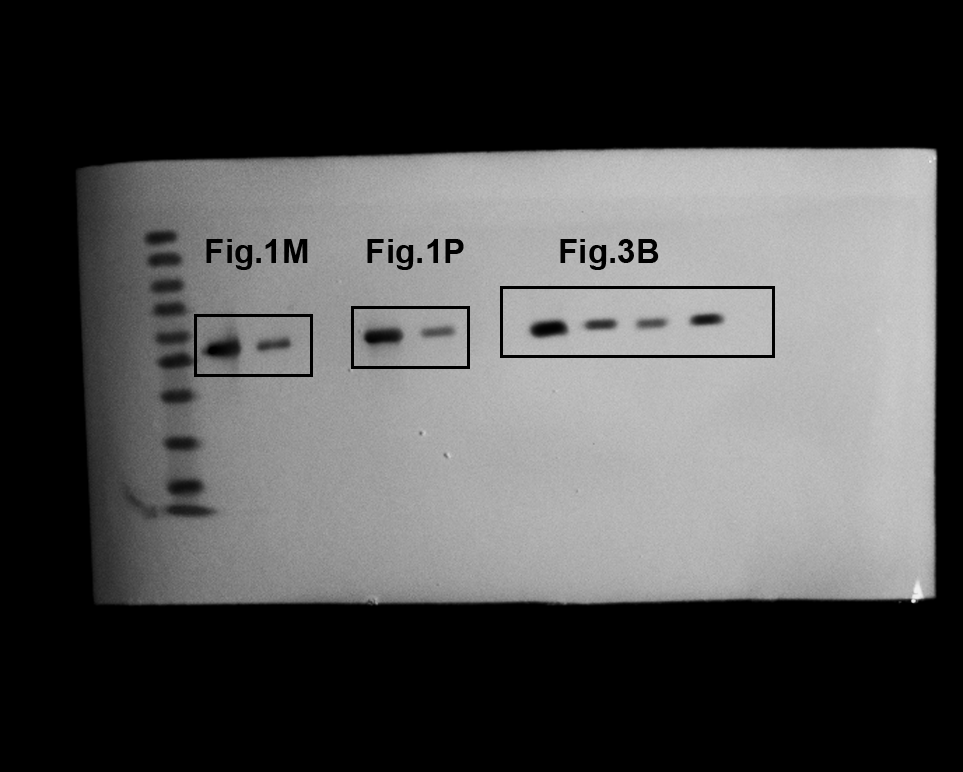 | |
| GAPDH | 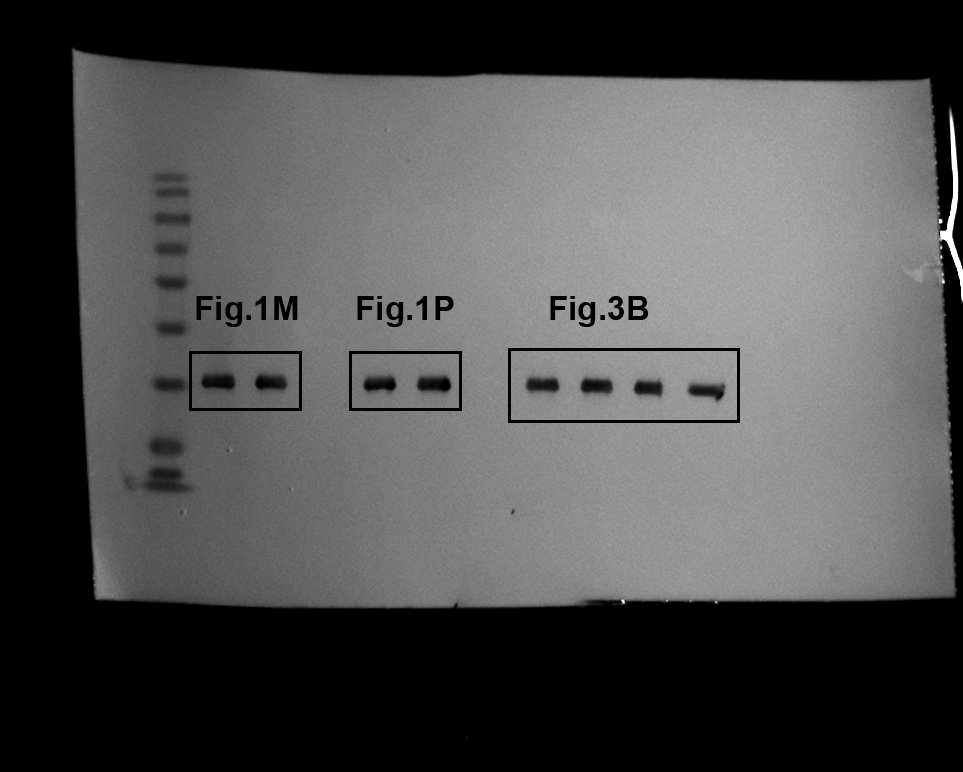 | |

Fig.2C

| Control | OGD/R | OGD/R+Vector | OGD/R+TRIM59 |
| --- | --- | --- | --- |
| 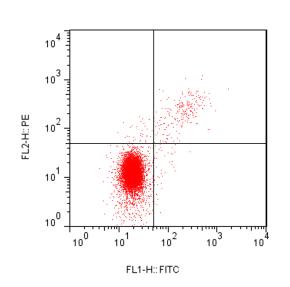 | 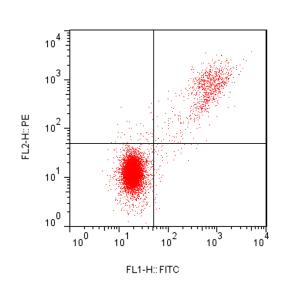 | 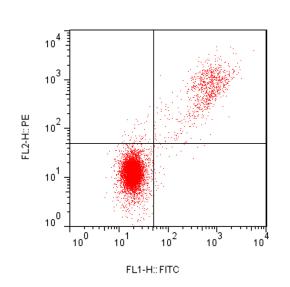 | 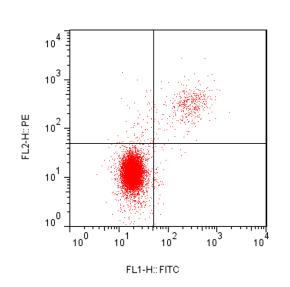 |

Fig.2H

|  | Control | OGD/R | OGD/R+Vector | OGD/R+TRIM59 |
| --- | --- | --- | --- | --- |
| NLRP3 | 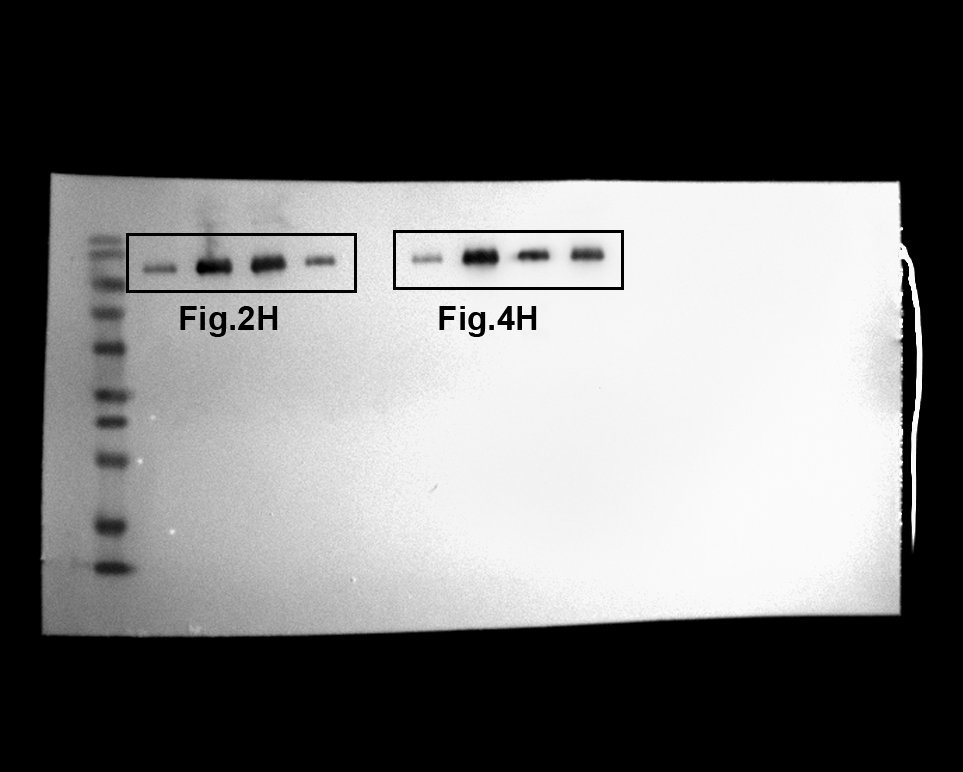 | | | |
| Cleavage-caspase-1 | 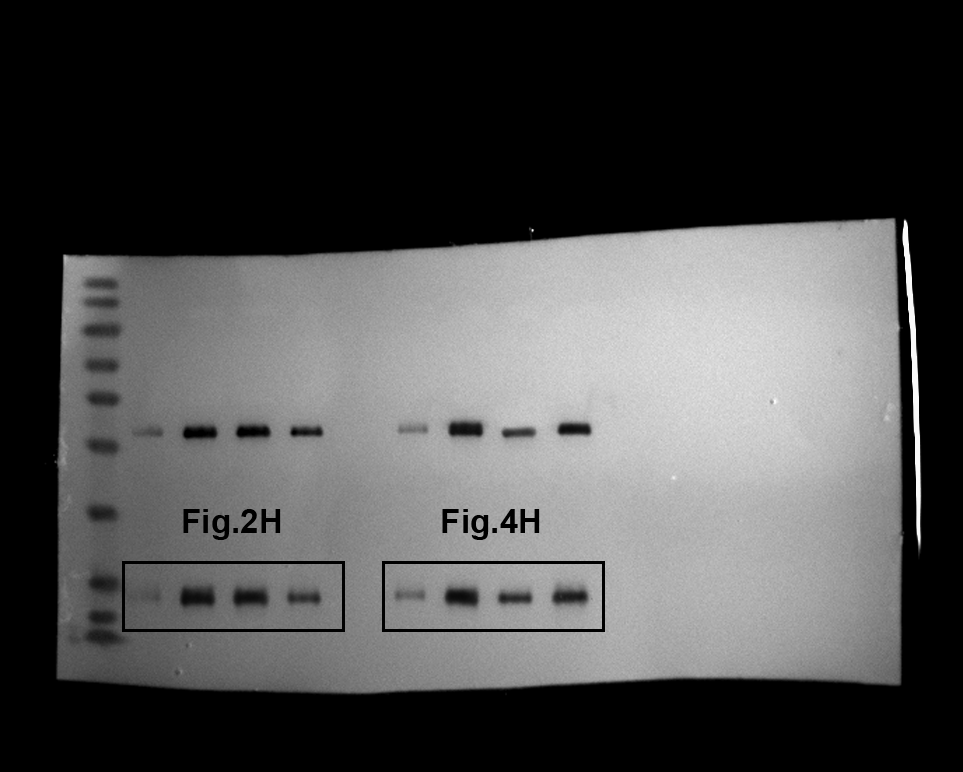 | | | |
| GSDMD-N | 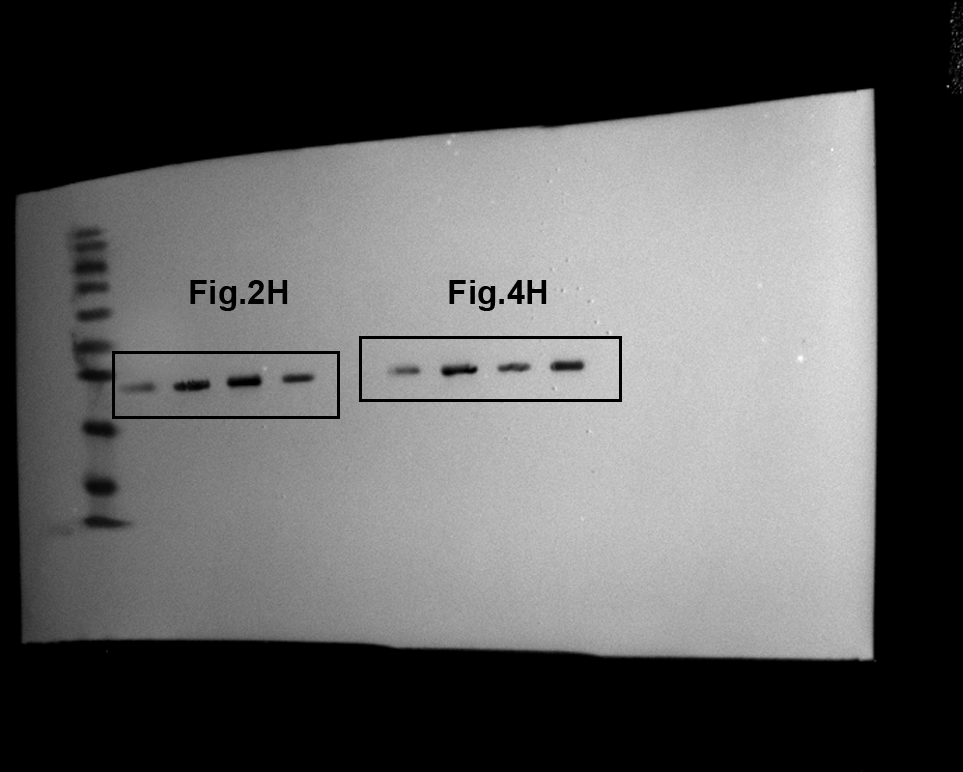 | | | |
| GAPDH | 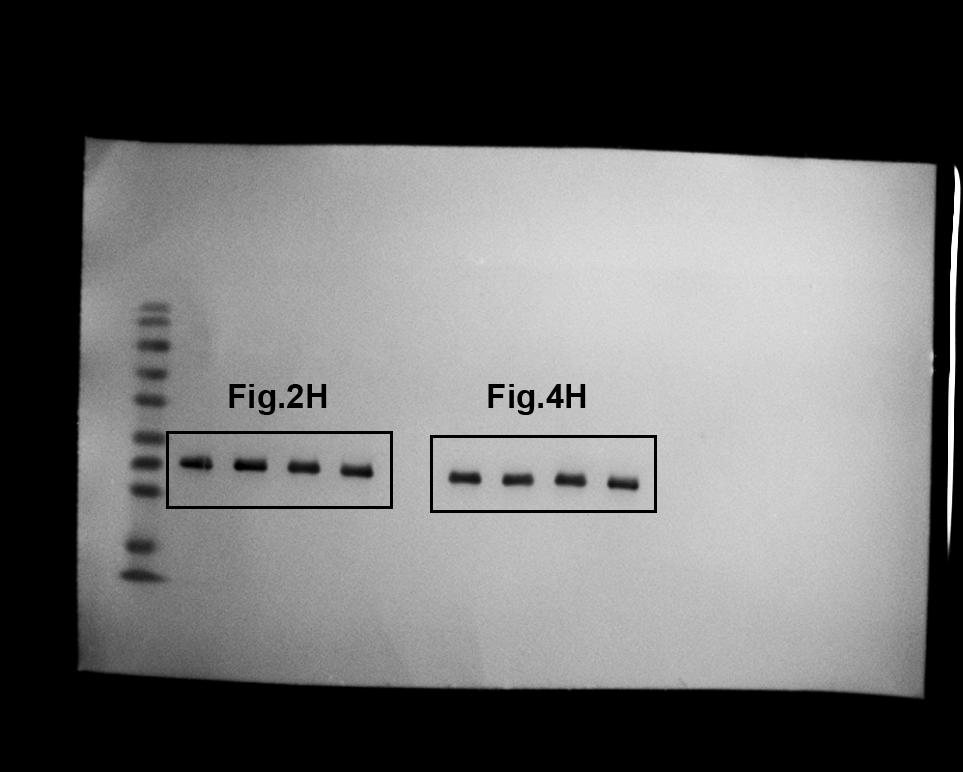 | | | |

Fig.3A

|  | IP:TRIM59 | | |
| --- | --- | --- | --- |
|  | Input | IgG | IP |
| TRIM59 | 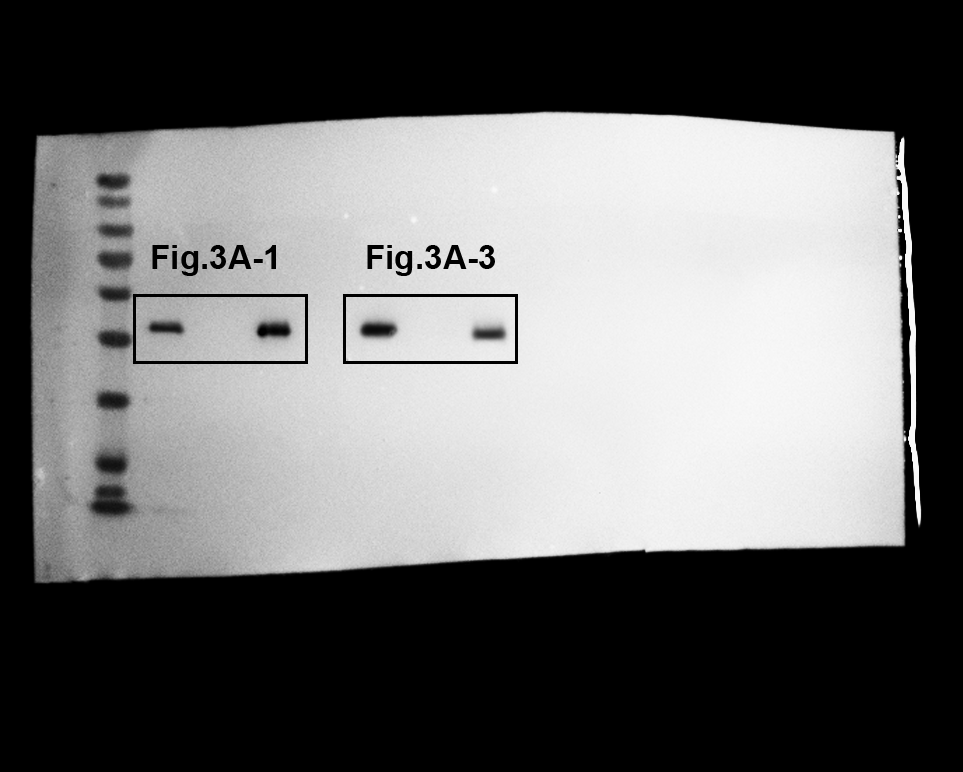 | | |
| NLRP3 | 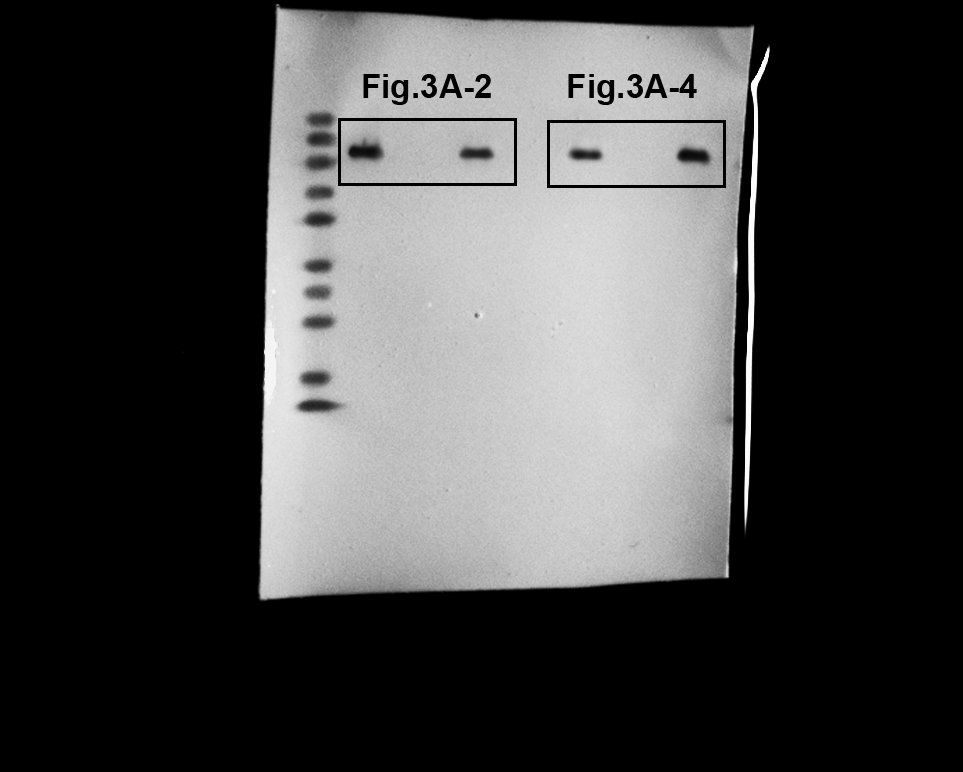 | | |

|  | IP:NLRP3 | | |
| --- | --- | --- | --- |
|  | Input | IgG | IP |
| TRIM59 | 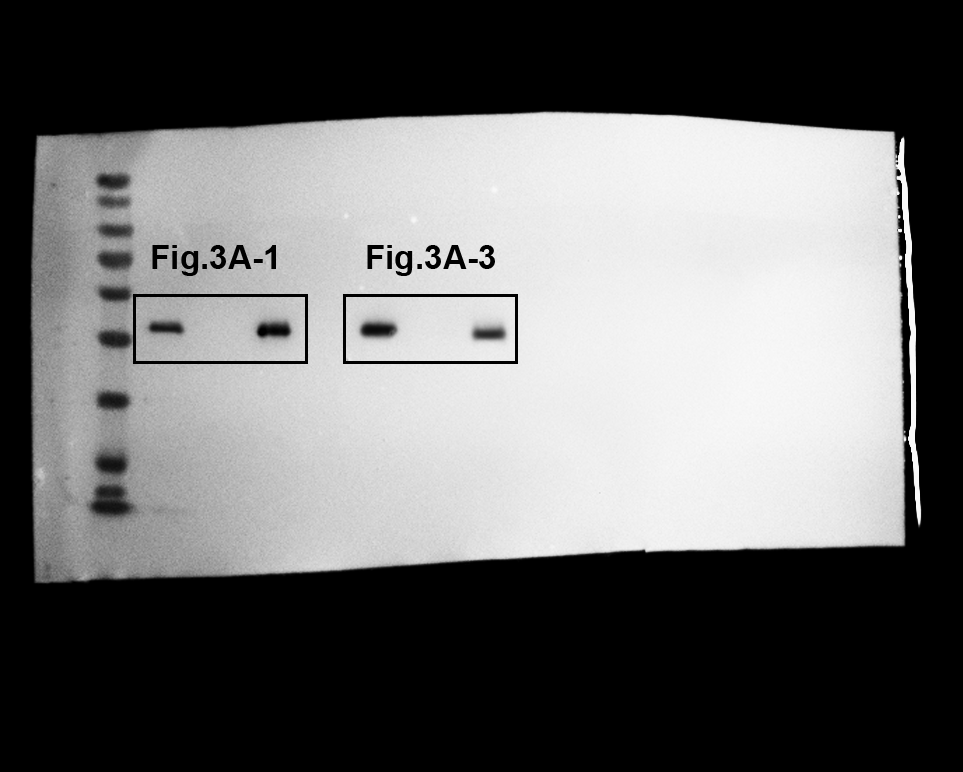 | | |
| NLRP3 | 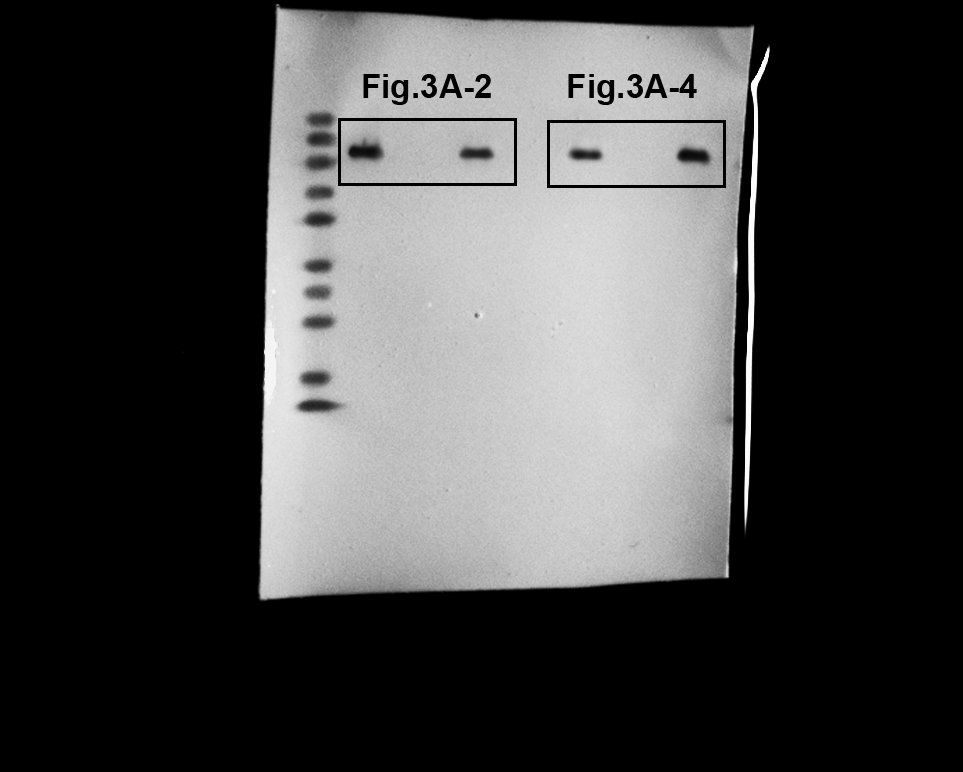 | | |

FiG.3B

| Flag-NLRP3 | + | + | + | + |
| --- | --- | --- | --- | --- |
| HA-UB | + | + | + | + |
| OGD/R | - | + | + | + |
| Vector | - | - | + | - |
| TRIM59 | - | - | - | + |
| IP:Flag  IB:HA | 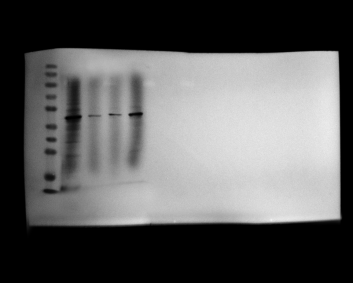 | | | |
| TRIM59 | 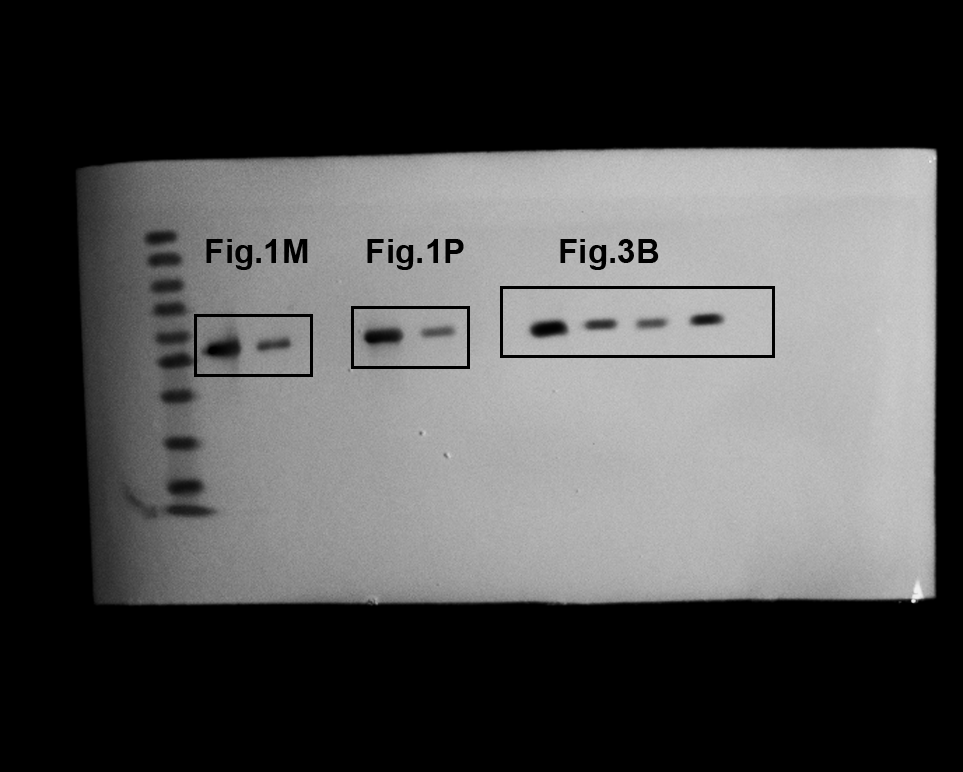 | | | |
| NLRP3 | 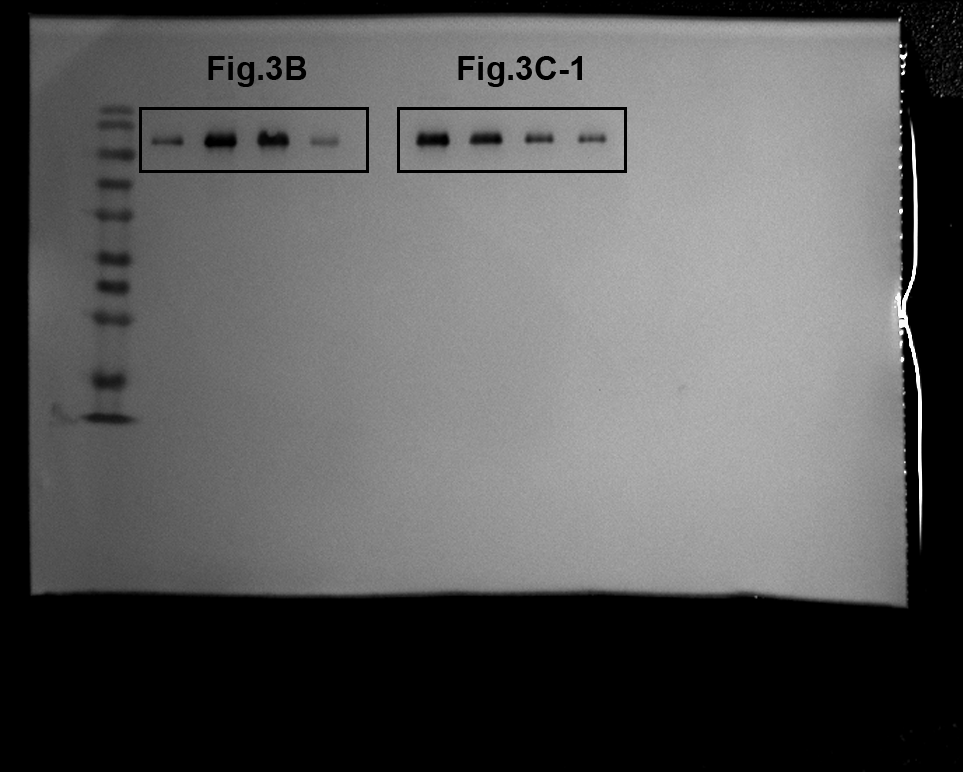 | | | |
| GAPDH | 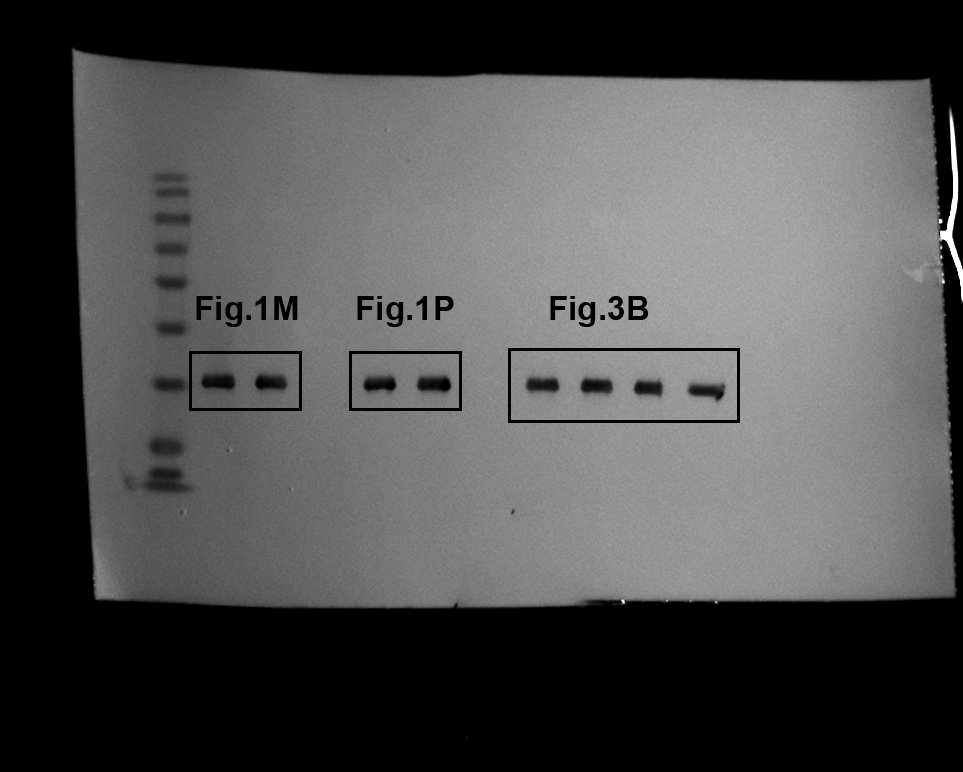 | | | |

Fig.3C

| Control | 0 | 2 | 4 | 8 CHX(h) |
| --- | --- | --- | --- | --- |
| NLRP3 | 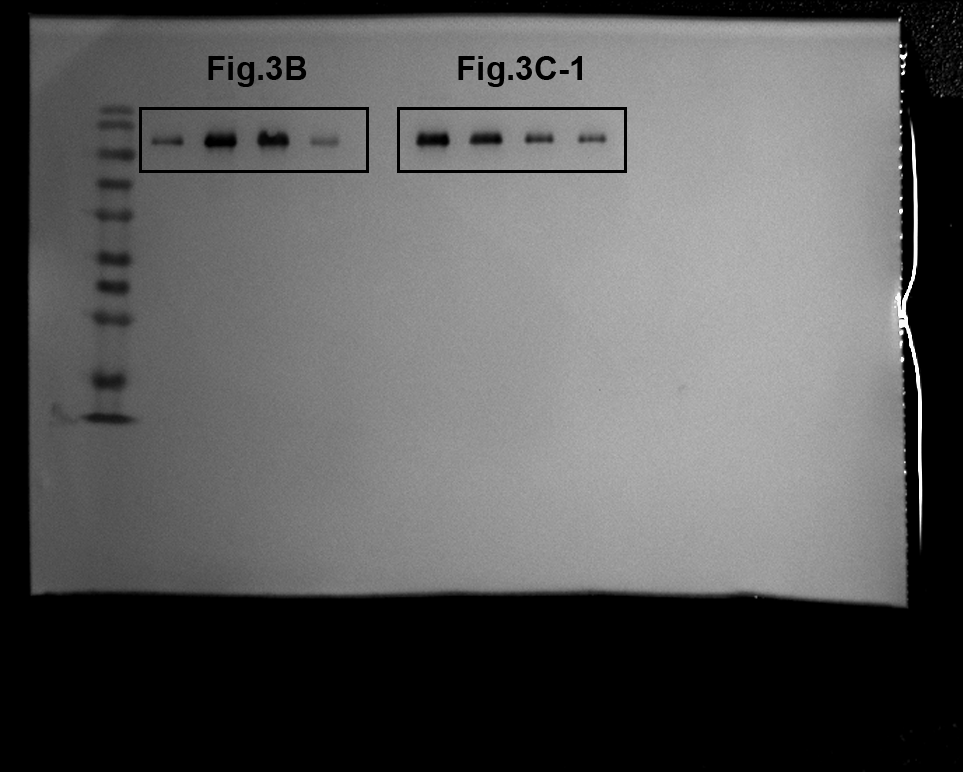 | | | |
| GAPDH | 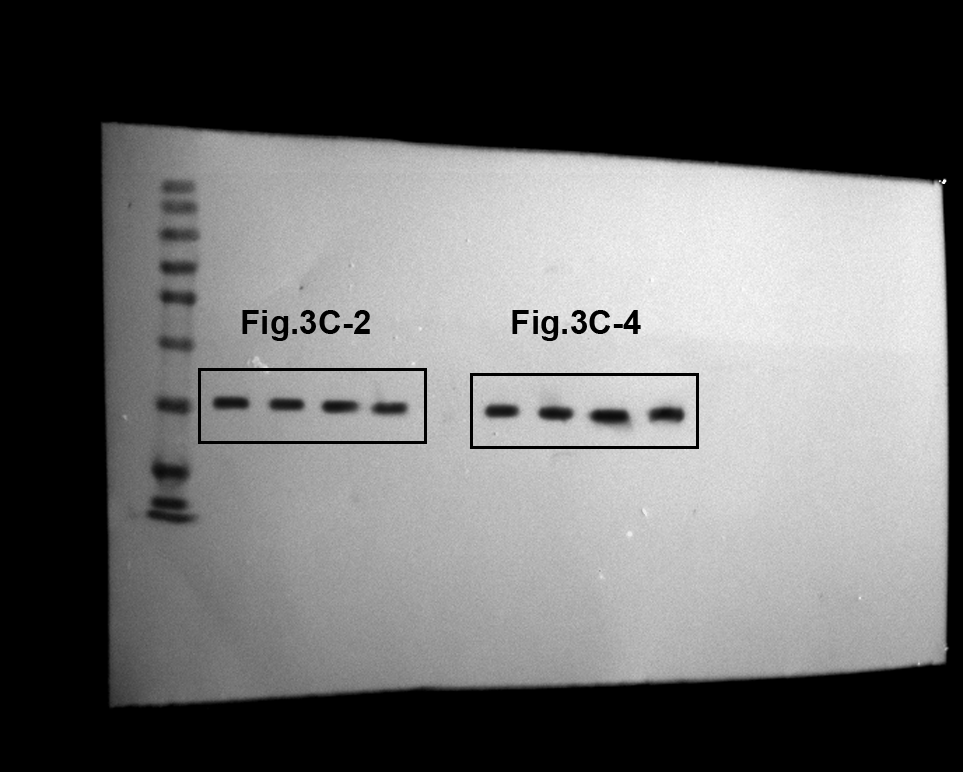 | | | |
| OGD/R | 0 | 2 | 4 | 8 CHX(h) |
| NLRP3 | 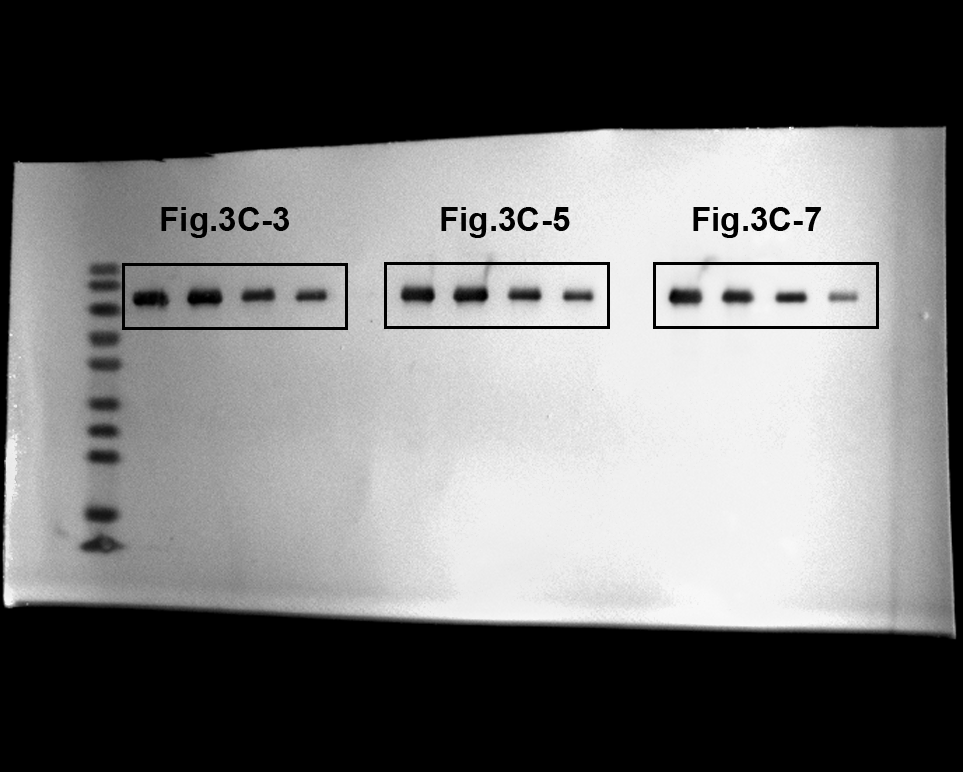 | | | |
| GAPDH | 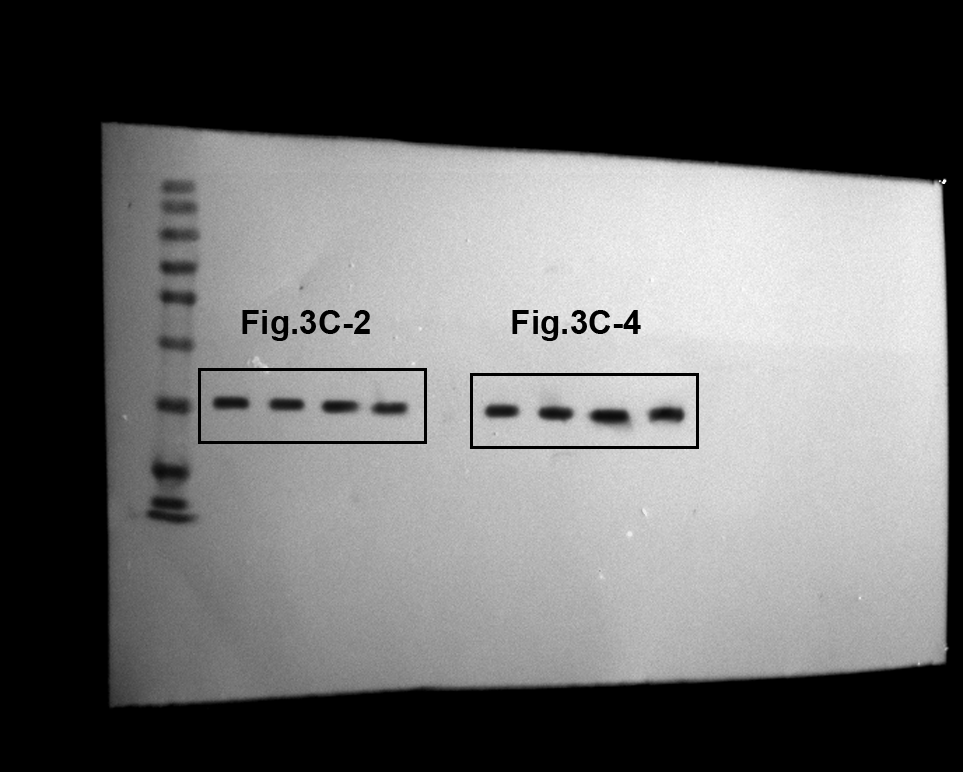 | | | |
| OGD/R+Vector | 0 | 2 | 4 | 8 CHX(h) |
| NLRP3 | 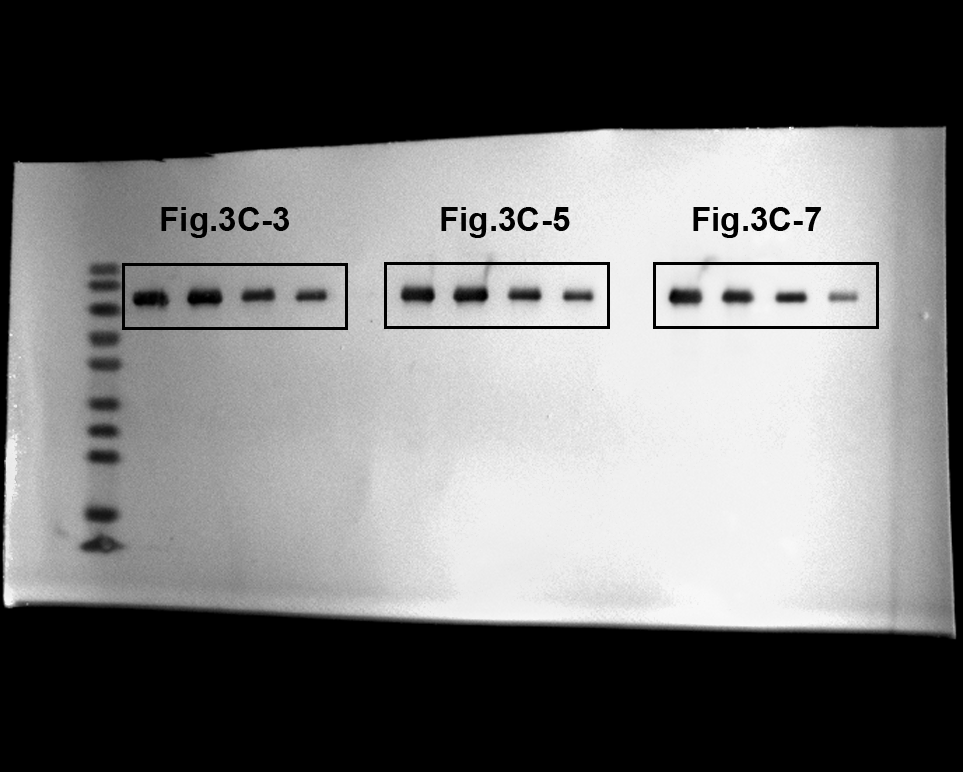 | | | |
| GAPDH | 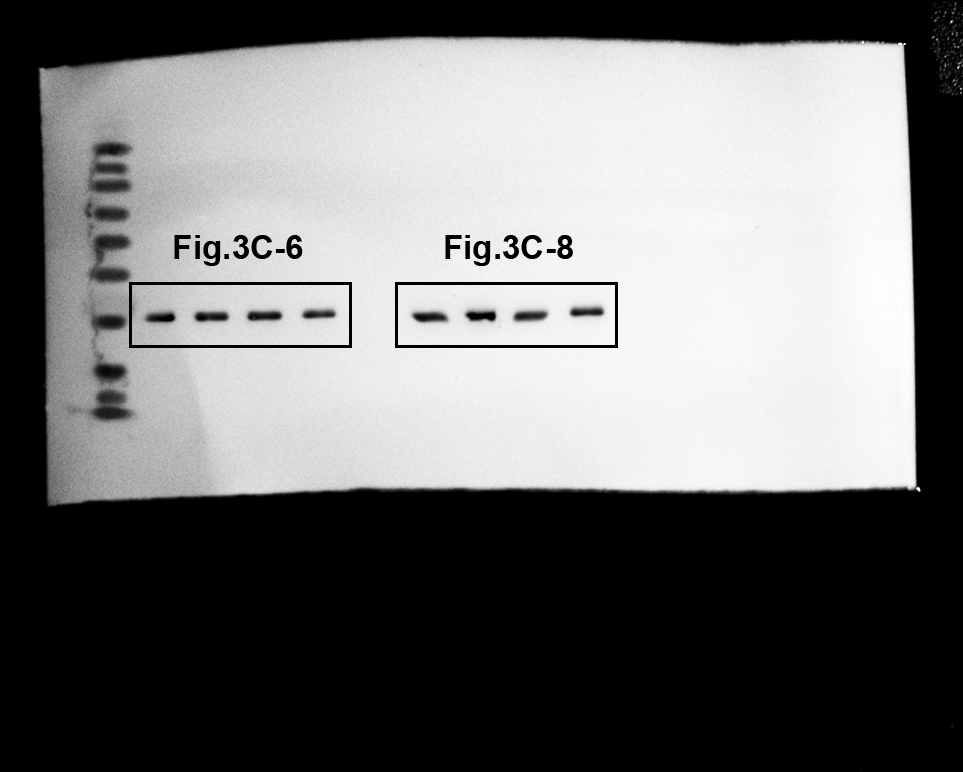 | | | |
| OGD/R+TRIM59 | 0 | 2 | 4 | 8 CHX(h) |
| NLRP3 | 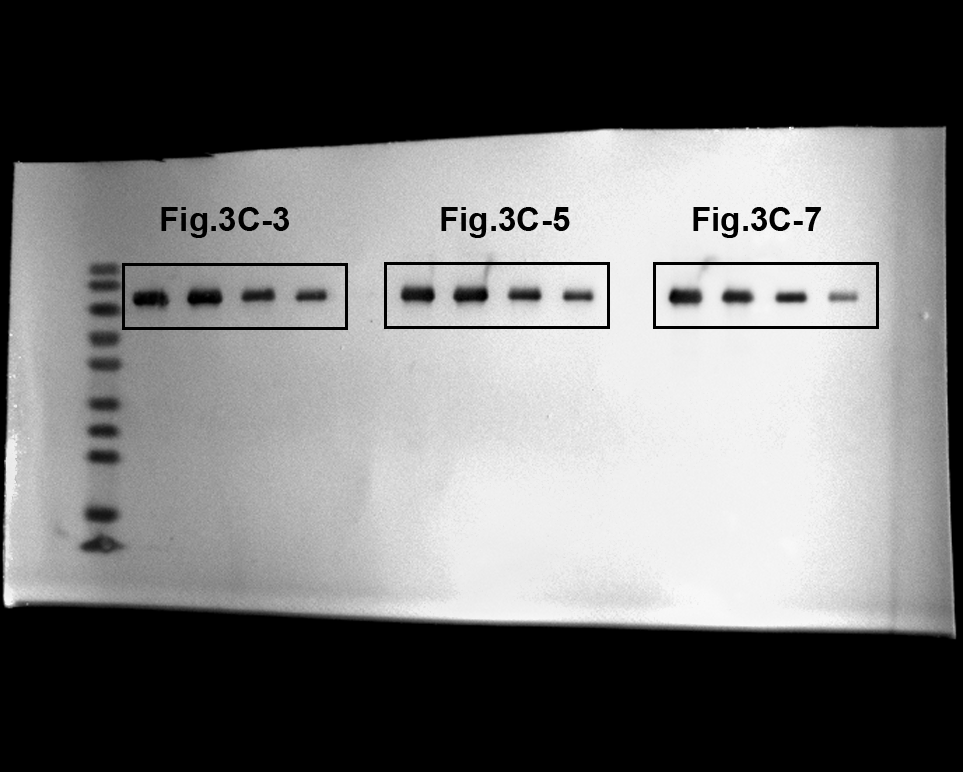 | | | |
| GAPDH | 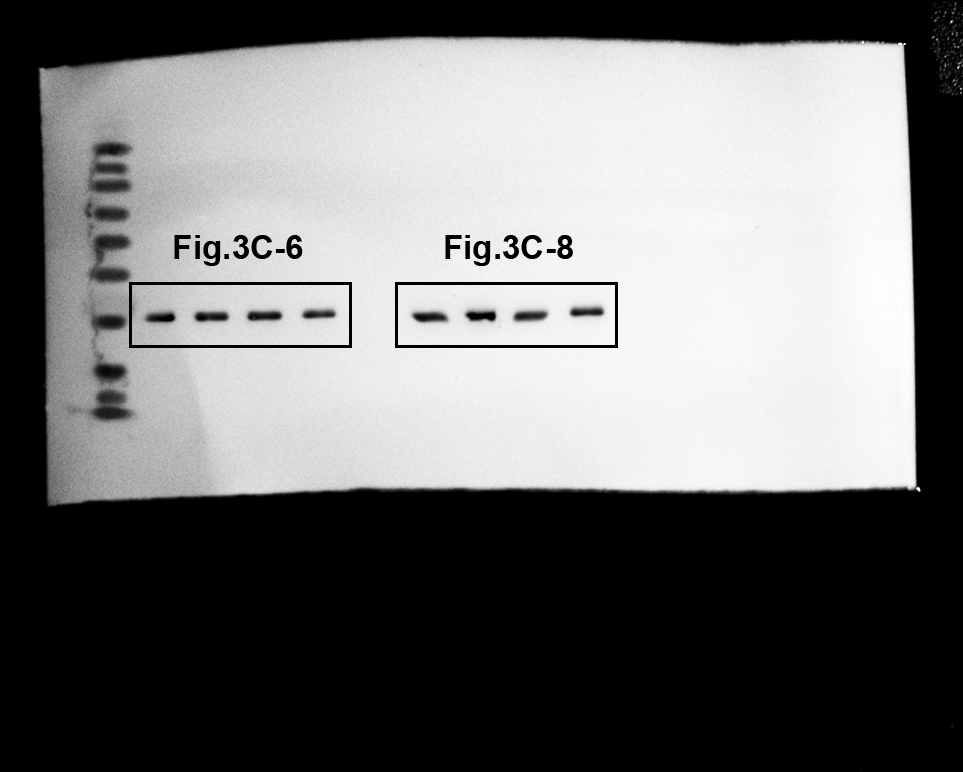 | | | |

Fig.4C

| Control | OGD/R | OGD/R+TRIM59+Vector | OGD/R+TRIM59+NLRP3 |
| --- | --- | --- | --- |
| 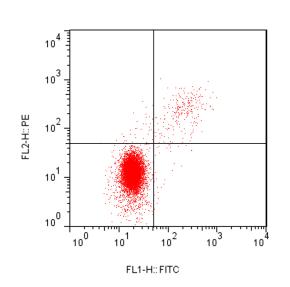 | 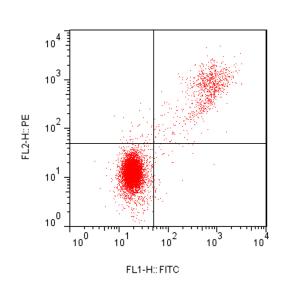 | 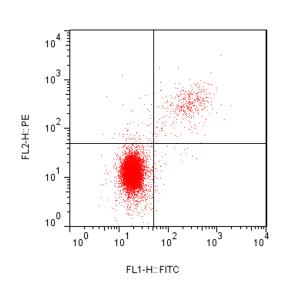 | 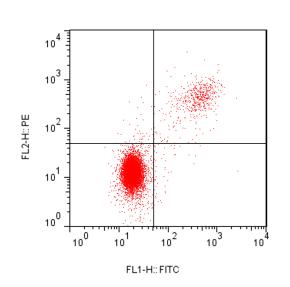 |

Fig.4H

|  | Control | OGD/R | OGD/R+TRIM59+Vector | OGD/R+TRIM59+NLRP3 |
| --- | --- | --- | --- | --- |
| NLRP3 | 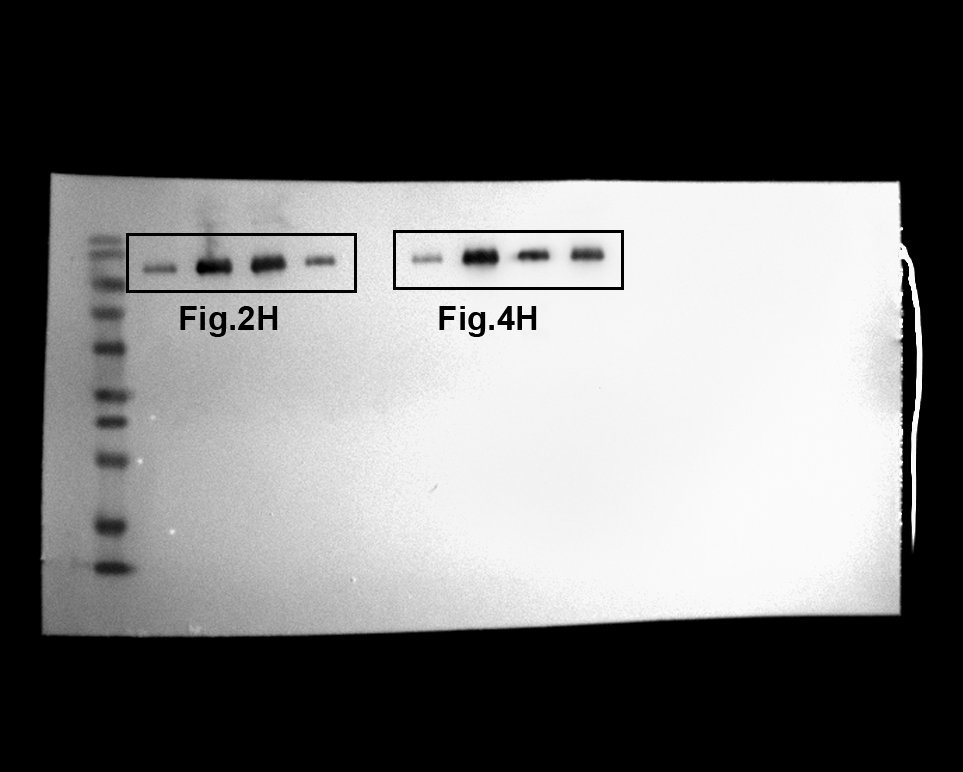 | | | |
| Cleavage-caspase-1 | 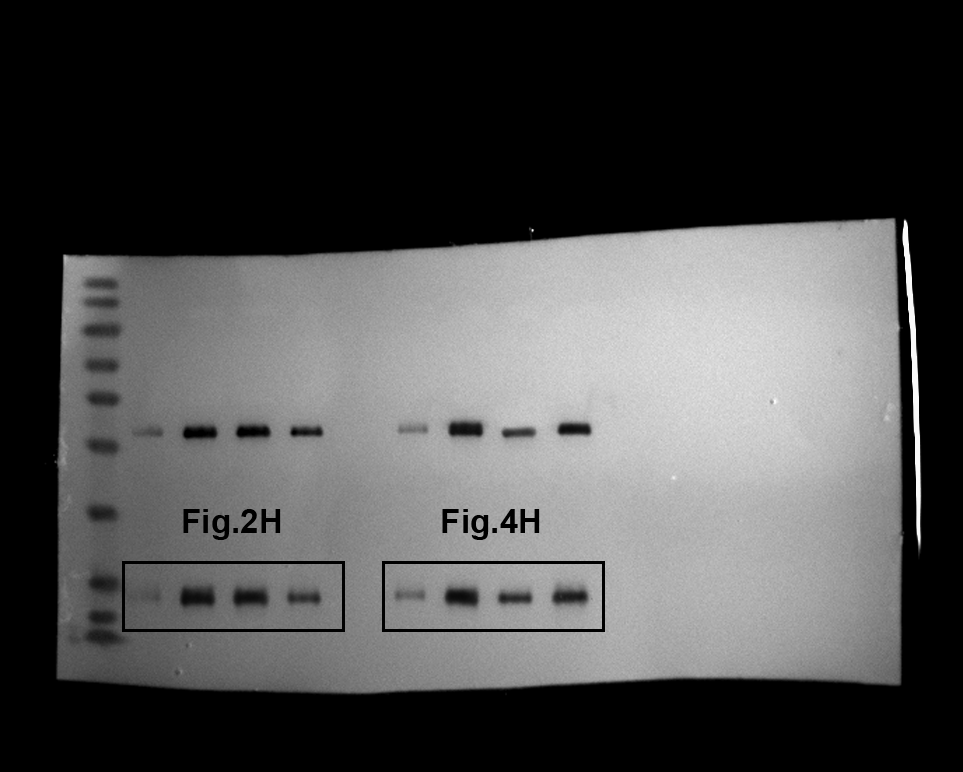 | | | |
| GSDMD-N | 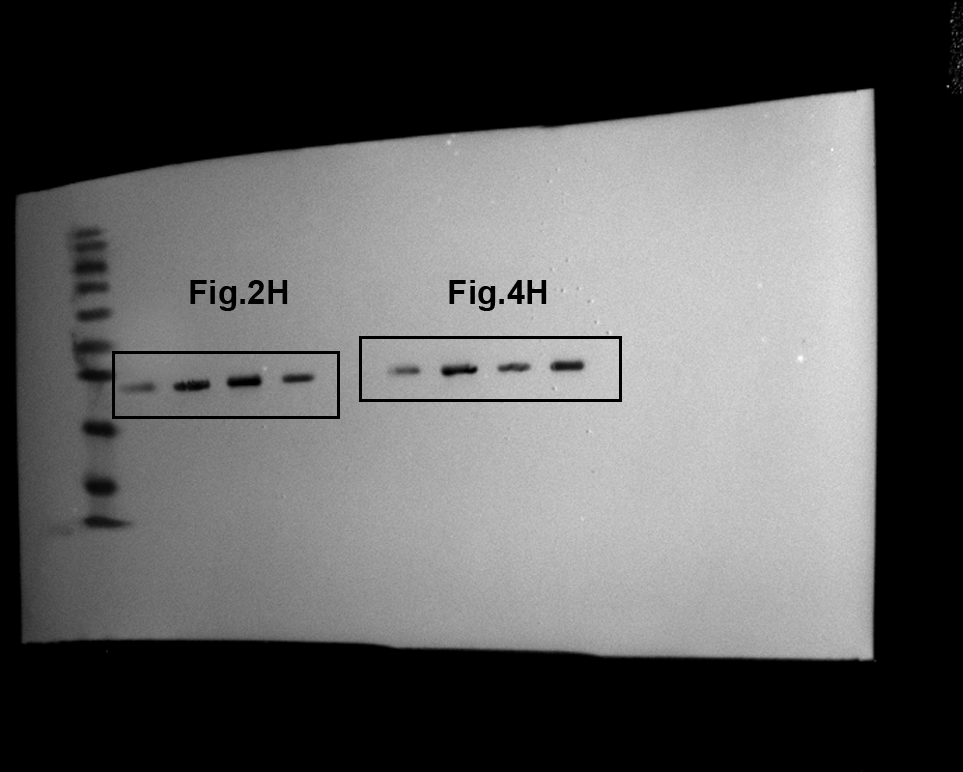 | | | |
| GAPDH | 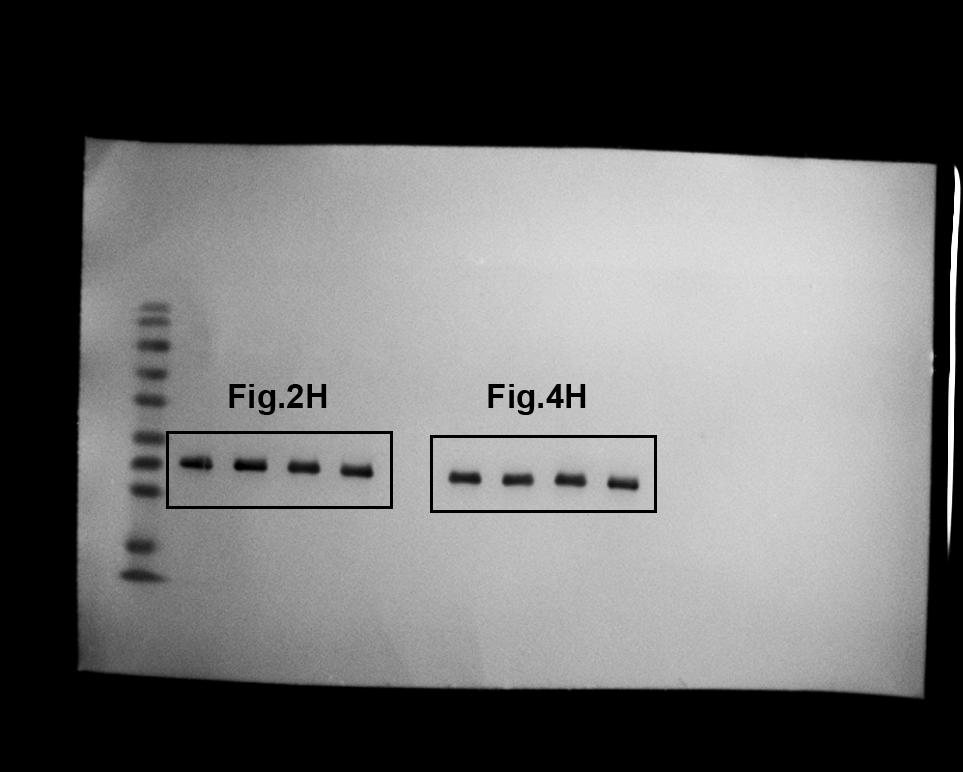 | | | |

Fig.5A

| Sham | MCAO | MCAO+LV-NC | MCAO+LV-TRIM59 |
| --- | --- | --- | --- |
| 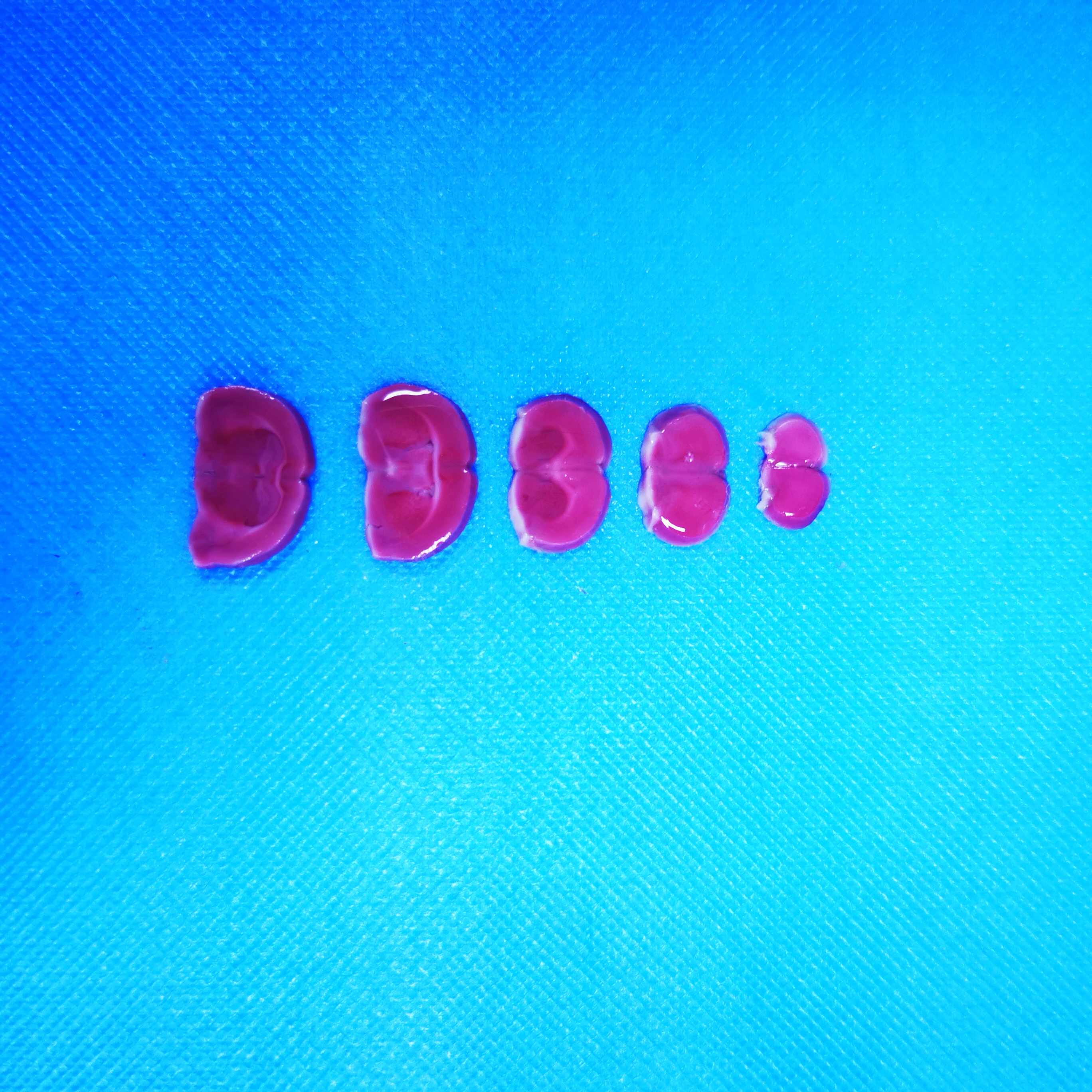 | 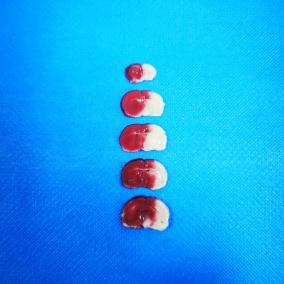 | 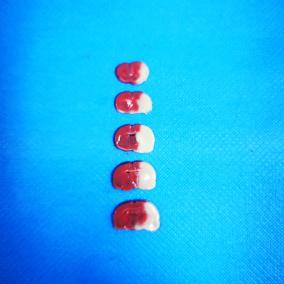 | 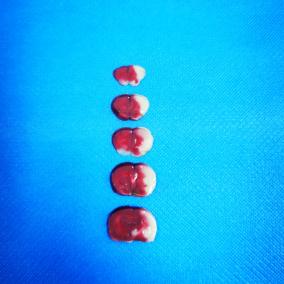 |

Fig.5H

|  | Sham | MCAO | MCAO+LV-NC | MCAO+LV-TRIM59 |
| --- | --- | --- | --- | --- |
| DAPI | 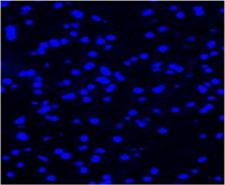 | 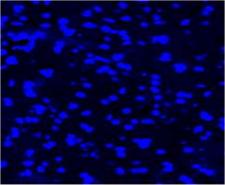 | 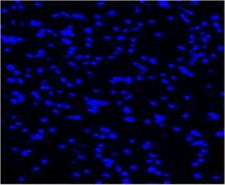 | 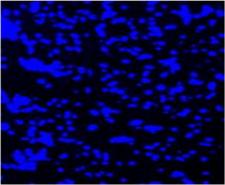 |
| IBA1 | 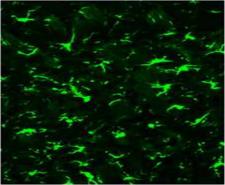 | 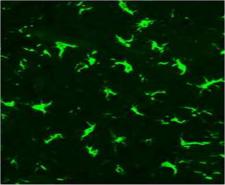 | 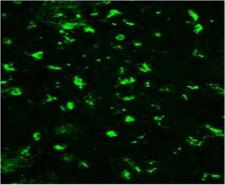 | 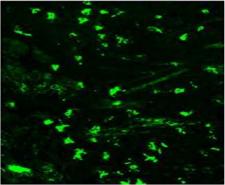 |
| NLRP3 | 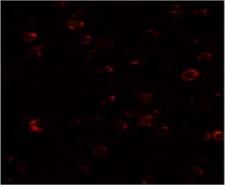 | 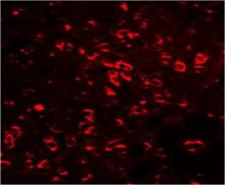 | 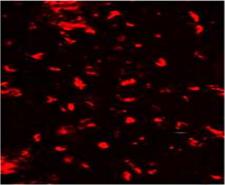 | 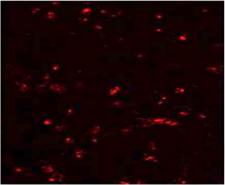 |
| Merge | 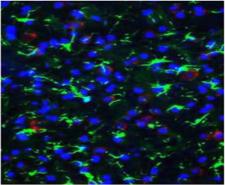 | 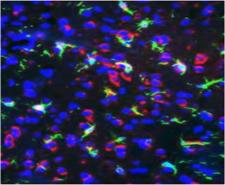 | 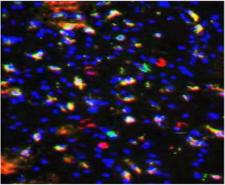 | 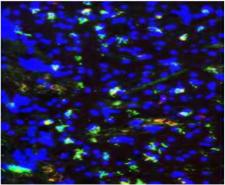 |
